# Ambulatory knee biomechanics and muscle activity 2 years after ACL surgery: InternalBrace^TM^-augmented ACL repair versus ACL reconstruction versus healthy controls

**DOI:** 10.1186/s12891-023-06916-7

**Published:** 2023-10-04

**Authors:** Linda Bühl, Sebastian Müller, Corina Nüesch, Katherine A. Boyer, Erica Casto, Annegret Mündermann, Christian Egloff

**Affiliations:** 1 Department of Orthopaedics and Traumatology, University Hospital Basel, Spitalstrasse 21, Basel, 4031 Switzerland; 2 Department of Biomedical Engineering, University of Basel, Allschwil, 4123 Switzerland; 3 Department of Clinical Research, University of Basel, Basel, 4031 Switzerland; 4 Department of Spine Surgery, University Hospital Basel, Basel, 4031 Switzerland; 5 Department of Kinesiology, University of Massachusetts Amherst, Amherst, MA 01003 USA; 6 Department of Orthopedics and Physical Rehabilitation, University of Massachusetts Medical School, Worcester, MA 01655 USA

**Keywords:** Anterior cruciate ligament, Hamstring autograft, Kinematics and kinetics, Muscle activation, Primary repair

## Abstract

**Background:**

Little is known about knee mechanics and muscle control after augmented ACL repair. Our aim was to compare knee biomechanics and leg muscle activity during walking between the legs of patients 2 years after InternalBrace^TM^-augmented anterior cruciate ligament repair (ACL-IB) and between patients after ACL-IB and ACL reconstruction (ACL-R), and controls.

**Methods:**

Twenty-nine ACL-IB, 27 sex- and age-matched ACL-R (hamstring tendon autograft) and 29 matched controls completed an instrumented gait analysis. Knee joint angles, moments, power, and leg muscle activity were compared between the involved and uninvolved leg in ACL-IB (paired t-tests), and between the involved legs in ACL patients and the non-dominant leg in controls (analysis of variance and posthoc Bonferroni tests) using statistical parametric mapping (SPM, P < 0.05). Means and 95% confidence intervals (CI) of differences in discrete parameters (DP; i.e., maximum/minimum) were calculated.

**Results:**

Significant differences were observed in ACL-IB only in minimum knee flexion angle (DP: 2.4°, CI [-4.4;-0.5]; involved > uninvolved) and maximum knee flexion moment during stance (-0.07Nm/kg, CI [-0.13;-0.00]; involved < uninvolved), and differences between ACL-IB and ACL-R only in maximum knee flexion during swing (DP: 3.6°, CI [0.5;7.0]; ACL-IB > ACL-R). Compared to controls, ACL-IB (SPM: 0–3%GC, P = 0.015; 98–100%, P = 0.016; DP: -6.3 mm, CI [-11.7;-0.8]) and ACL-R (DP: -6.0 mm, CI [-11.4;-0.2]) had lower (maximum) anterior tibia position around heel strike. ACL-R also had lower maximum knee extension moment (DP: -0.13Nm/kg, CI [-0.23;-0.02]) and internal knee rotation moment (SPM: 34–41%GC, P < 0.001; DP: -0.03Nm/kg, CI [-0.06;-0.00]) during stance, and greater maximum semitendinosus activity before heel strike (DP: 11.2%maximum voluntary contraction, CI [0.1;21.3]) than controls.

**Conclusion:**

Our results suggest comparable ambulatory knee function 2 years after ACL-IB and ACL-R, with ACL-IB showing only small differences between legs. However, the differences between both ACL groups and controls suggest that function in the involved leg is not fully recovered and that ACL tear is not only a mechanical disruption but also affects the sensorimotor integrity, which may not be restored after surgery. The trend toward fewer abnormalities in knee moments and semitendinosus muscle function during walking after ACL-IB warrants further investigation and may underscore the importance of preserving the hamstring muscles as ACL agonists.

**Level of evidence:**

Level III, case-control study.

**Trial registration:**

clinicaltrials.gov, NCT04429165 (12/06/2020).

**Supplementary Information:**

The online version contains supplementary material available at 10.1186/s12891-023-06916-7.

## Introduction

Anterior cruciate ligament (ACL) tears can lead to knee instability [1] and – even after gold standard surgery of ACL reconstruction (ACL-R) – to impaired knee mechanics [2, 3], muscle function [4], coordination [5], and osteoarthritis [6]. In recent years, there has been a resurgence of interest in primary ACL repair, which had been abandoned in favour of ACL-R in the past [7], with the aim of achieving better results. In this context, ACL repair with additional synthetic tape augmentation using the InternalBrace™ (ACL-IB, Arthrex Inc., USA) has been introduced for proximal ACL tears [8]. In ACL-IB, the InternalBrace^TM^-augmentation consists of a polyethylene tape that is placed on the femorally reattached native ACL and fixed to the femur and the tibia [8].

The advantages of ACL repair procedures (augmented or non-augmented) over traditional ACL-R (where the torn ligament is replaced with an autologous muscle tendon [9]) are a less invasive procedure with preserved muscle-tendon units (no graft harvesting) and preservation of native ACL fibres, including their native tibial origin. Functionally, ACL repair is thought to restore proprioception and natural knee mechanics, potentially reducing the risk of secondary knee osteoarthritis [8, 10–12]. While comparable patient-reported outcomes [13–16], higher, non-inferior or lower anterior knee laxity [13–16] and comparable pivot-shift test results [14, 15] have been reported in clinical exams after ACL-IB versus ACL-R, studies of the presumed mechanical benefit during motion tasks after augmented ACL repair are lacking [17]. This raises the question of whether, in addition to the surgical advantages, ACL-IB also results in a good functional-biomechanical outcome and whether this outcome is comparable to that in patients after the gold standard ACL-R. As the muscles spanning the knee joint can influence knee joint mechanics [18, 19] and ACL loading [20, 21], both biomechanical and muscle activity parameters must be considered to gain a comprehensive insight into the knee joint behaviour during locomotion.

The aim of this study was to compare knee biomechanics and leg muscle activity during walking.

- between the legs of patients 2 years after ACL-IB (side-to-side difference (SSD) in ACL-IB), and
- between the involved leg of patients 2 years after ACL-IB, the involved leg of patients 2 years after ACL-R, and the non-dominant leg of healthy controls (leg difference between groups).

Considering the less invasive procedure and greater preservation of natural knee joint structures in ACL-IB, we expect no SSD in patients after ACL-IB (first hypothesis) and more natural (closer to control) biomechanics after ACL-IB than after ACL-R (second hypothesis).

## Methods

This study is a substudy of a larger non-randomised comparative umbrella study with retrospective data collection [22].

### Participants

We enrolled patients who underwent ACL surgery at our institution. Inclusion criteria for patients were 2 years since primary InternalBrace^TM^-augmented ACL repair after proximal rupture (Sherman classification type I and II [23]) performed within a maximum of 5 weeks after the index injury, or 2 years since primary single-bundle ACL reconstruction with autologous hamstring tendon (semitendinosus and, if required (graft width < 7 mm), gracilis tendon) performed within a maximum of 8 months after the index injury. The maximum time from injury to surgery for ACL-IB was chosen because primary ACL repair is recommended in the early phase after rupture [8, 24]. The maximum time from injury to surgery for ACL-R was chosen to reduce the influence of manifested deviations due to previous periods of conservative treatment. Exclusion criteria for patients were concomitant rupture of the posterior cruciate ligament, complete rupture of both collateral ligaments, and previous injury or surgery in the involved or uninvolved leg. Inclusion criteria for controls were no history of knee injury. Inclusion criteria for all participants were age between 18 and 60 years, a body mass index of < 35 kg/m^2^, no known neuromuscular pathology, and the ability to give informed consent. Hospital cases of InternalBrace^TM^-augmented ACL repair surgery and ACL reconstruction surgery were screened for eligible patients 2 years after ACL-IB and ACL-R according to our inclusion and exclusion criteria. Controls were recruited from the local area. Each ACL-IB patient was sex-matched to a patient after ACL-R and a control subject with a maximum age difference of no more than 4 years between all groups [22].

Twenty-nine patients after unilateral primary ACL-IB, 27 sex- and age-matched patients after ACL-R with hamstring autograft (23 with semitendinosus tendon; four with semitendinosus and gracilis tendon), and 29 sex- and age-matched healthy controls were included (Table 1). To complete our ACL-R group and ensure an appropriate age and sex matching, we deviated from the published protocol [22] and recruited five patients who had received a hamstring autograft from two other medical centres. All patients had a complete ACL tear. As reported in previous studies related to the umbrella study project [22, 25, 26], anthropometry, timing of follow-up, and activity level (Tegner Activity Score (TAS) [27]) did not differ between groups (Table 1). Surgery for primary augmented ACL repair was performed 8 days earlier after the index injury than for primary ACL-R, because ACL-IB is performed early after injury to allow for healing [8, 24].

**Table 1:** Characteristics of patients after InternalBrace^TM^-augmented anterior cruciate ligament repair (ACL-IB), ACL reconstruction (ACL-R) and controls

*Parameter*	*ACL-IB* *(n = 29)*	*ACL-R* *(n = 27)*	*Controls* *(n = 29)*	*P value*
Sex (N, male/female)	13/16	13/14	13/16	
Age (years)	36.8 (10.6)	37.0 (10.7)	37.0 (10.7)	0.995
Body mass (kg)	73.2 (10.9)	73.1 (14.5)	70.2 (16.6)	0.656
Body height (cm)	172.2 (7.8)	170.5 (7.4)	172.6 (10.8)	0.643
Body mass index (kg/m²)	25.5 [21.2;26.5]*	24.5 [21.9;27.5]*	23.1 [20.4;24.9]*	0.134*
Time injury to surgery (days)	20 [15;25]*	28 [14;56]*		0.026*
Follow-up (months)	24.4 [23.6;27.2]*	24.2 [23.6;24.9]*		0.184*
TAS at follow-up	4.0 [4.0;6.0]*	4.0 [4.0;5.0]*	4.0 [3.0;5.0]*	0.329*

TAS, Tegner Activity Score

distributions are given as number of subjects and percentage of the respective group; values are given as mean (standard deviation) and p-values for one-way analysis of variance

*values are given as median [25;75] percentile and p-values for Kruskal Wallis or Mann-Whitney-U tests because quantile-quantile-plots revealed no normal distribution

bold printed values indicate a significant difference between groups, p < 0.05

### Procedures

All participants were examined in a single visit to the Laboratory of Functional Biomechanics at the University Hospital Basel (Switzerland). Anthropometric parameters were measured including all parameters required for the underlying gait models (e.g., height, leg length), and participants were prepared for the subsequent gait analysis. Participants were then asked to walk in their own shoes at self-selected speeds along a 10-m walkway with two embedded force plates until at least four valid trials per leg were obtained (i.e., foot isolated on a force plate). Anthropometric data were managed using REDCap© [28, 29]. Gait data were processed using MATLAB (R2020b, The MathWorks Inc., Natick, MA, US).

### Gait data

#### Knee biomechanics

**Fig. 1:**
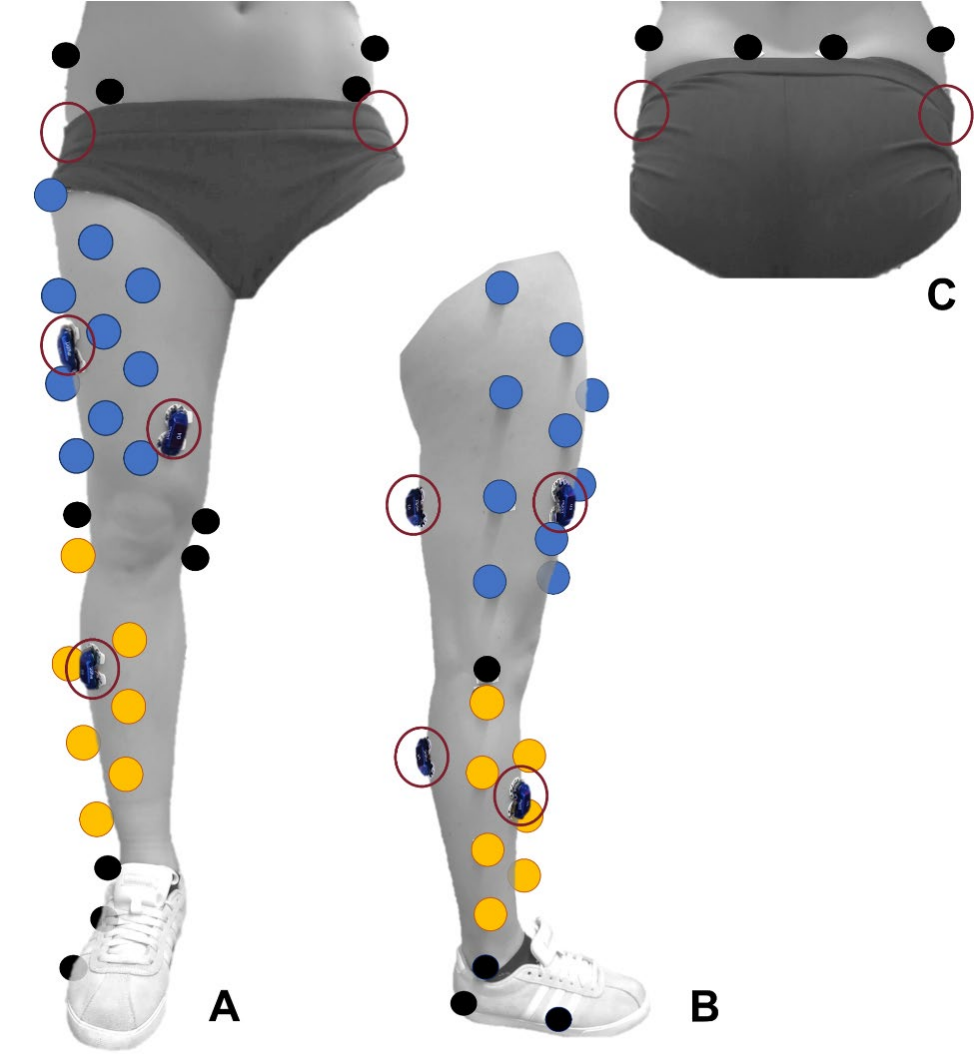
Placement of skin markers (including thigh (blue) and shank (yellow) clusters), and electromyographic electrodes (red circles, electrodes at the pelvis are placed on the skin under the shorts) in the frontal (A), lateral (B) and posterior (C) view. (Cluster marker: thigh (blue); shank (yellow); electromyography electrodes (red circles, electrodes at the hip for gluteus medius under the shorts)). Figure retrieved and modified from the RetroBRACE study protocol [22]. © Author(s) 2022. Re-use permitted under CC BY-NC

**Fig. 2:**
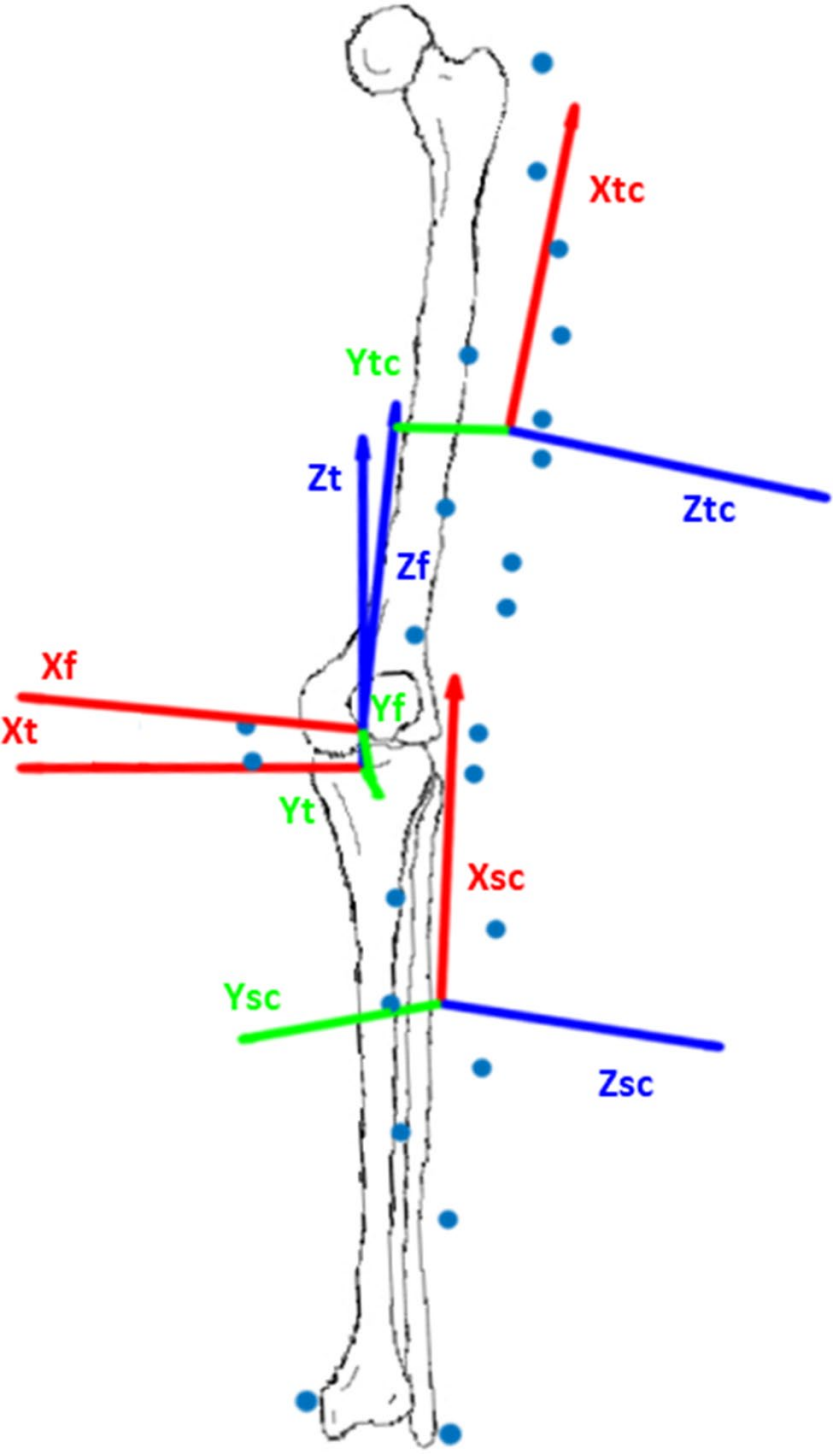
Anatomical (femur: f; tibia: t) and cluster coordinate systems (thigh: tc; shank: sc) for kinematic calculations. (Left leg. Origins: anatomic coordinate systems: midpoint of the medial and lateral epicondyles (femur) and medial and lateral tibia plateau (tibia); cluster coordinate system: center of mass of cluster markers. Axes: femur: temporary X-axis from lateral to medial epicondyles; Z-axis from lateral epicondyle to greater trochanter; tibia: temporary X-axis from lateral to medial tibia plateau; Z-axis from the midpoint between the lateral and medial malleoli to origin of tibia coordinate system. For each anatomical coordinate system, the Y-axis was defined as cross product between the Z- and temporary X-axis, and the X-axis as cross product between the Y- and Z-axis. Axes of the cluster coordinate system: eigenvectors and eigenvalues of the inertia tensor of the cluster markers assuming a unit weight for each marker (PCT approach))

Knee biomechanics (kinematics and kinetics) during walking were recorded using a motion capture system (VICON, Oxford, UK; sampling rate 240 Hz) and two force plates (Kistler 9260AA6, Kistler AG, Winterthur, Switzerland; sampling rate 2400 Hz). Reflective markers (Ø 16 mm) were placed on the skin at defined bony landmarks (pelvis, femur, tibia, fibula and foot), with additional nine and six cluster markers on the femur and the tibia, respectively (Fig. 1). The full set of markers was used to calibrate the subject-specific marker model (anatomical segment marker and cluster marker-based coordinate systems) in an upright, hip-width static reference position (Fig. 2). All medial markers were then removed.

Marker trajectories were processed (labelled, gap-filled, filtered with Woltring filter [30]) in Nexus (VICON, Oxford, UK). Knee kinematics (3D rotations and anterior tibia translation) were calculated using the point cluster technique (PCT) [31, 32]. In this approach, the cluster-based coordinate systems (femur: femur cluster and greater trochanter marker; tibia: tibia cluster and lateral tibia plateau marker, Fig. 1) and their relationship to the anatomical femur and tibia coordinate systems in the static trial were used to calculate the anatomical segments in the dynamic trials using the eigenvalue and eigenvector method of the inertia tensor of the clusters to correct for rigid anatomical segments (PCT approach, Fig. 2) [31, 32]. An Euler angle sequence (xyz) was used to calculate the knee angles between the anatomical femur and tibia coordinates system in the dynamic trials (Fig. 2), with the tibia as reference (distal segment). Anteroposterior translation was defined as the anterior position of the origin of the femur coordinate system (midpoint of the transepicondylar line) relative to the origin of the tibia coordinate system (midpoint medial and lateral tibia plateau, Fig. 2). These data were then inverted to show positive values as anterior tibia translation in relation to the femur. This method of analysis has previously been used in patients after ACL-R [33]. The conventional gait model (CGM) version 2.3 [34] was used for inverse kinetic calculations, with the middle markers on the lateral side, and the distal and proximal markers on the anterior side of the thigh and shank clusters were used as CGM cluster markers, respectively. Joint kinetics were filtered with a 4th -order low‐pass Butterworth filter (cut‐off frequency 12 Hz), and joint moments (expressed as external moments) and power were normalised to body mass (Nm/kg, W/kg). Positive values represented anterior tibia translation; flexion, abduction, and internal tibia rotation angles; flexion, adduction, and internal tibia rotation moments; and joint power generation. Gait events were detected from the force plate data using thresholds of ≥ 20 N for foot strike and < 20 N for foot‐off.

#### Muscle activity

Muscle activity of the gluteus medius, vastus lateralis, vastus medialis, semitendinosus, tibialis anterior and gastrocnemius medialis muscles was recorded using surface electromyography (EMG) electrodes (Myon AG, Schwarzenberg, Switzerland, sampling rate 2400 Hz, inter-electrode distance 25 mm). EMG electrodes were attached bilaterally to shaved (if necessary) and alcohol-cleaned skin according to the standards of the SENIAM project [35] (Surface Electromyography for the Non-Invasive Assessment of Muscles; Fig. 1).

Raw EMG data were filtered (4th -order bandpass filter, 20–450 Hz), full-wave rectified, and smoothed (4th -order lowpass filter, 6 Hz) [36]. EMG amplitudes were normalised per participant and per leg. Muscle activity of the thigh muscles (semitendinosus and vasti) were normalised to maximum voluntary isokinetic contraction (MVC), determined in two trials of four repetitions each at 60°/s on a dynamometer (Biodex Medical Systems, Shirley, USA) [22]. All other muscles were normalised to their maximum contraction during walking (maximum contraction walking, MCW) [4].

Trajectories of knee kinetics, kinematics and muscle activity data were time-normalised to the gait cycle (defined from foot strike to foot strike, 0–100% gait cycle (GC)) and averaged across trials for each participant and leg for further statistical analysis.

### Statistical analysis

Group characteristics, including anthropometric and clinical data and spatio-temporal parameters, were compared between groups using analysis of variance (ANOVA), and Kruskal-Wallis or Mann-Whitney U tests (patient data only) when quantile-quantile-plots did not yield a normal distribution. Based on our sample size estimation [22], 28 subjects were required to detect a statistically significant difference with 80% power and a significance level of 5%. Statistical analysis of group characteristics was performed in SPSS Version 28.0.1.0 (IBM Corporation, Amonk, NY, USA), and gait data were analysed using MATLAB (R2020b, The MathWorks Inc., Natick, MA, US).

Biomechanical and muscle activity patterns (i.e., spatio-temporal, kinematic or kinetic trajectories) are commonly described and analysed using discrete parameters computed from the respective time series data (i.e., local extrema or value at initial contact) [2, 3] or through time series analysis, such as statistical parametric mapping (SPM). While time series analysis allows for a more comprehensive analysis of biomechanical data trajectories [37], when comparing biomechanical data between multiple trials (i.e., of legs or groups) it requires that the datasets are normalised to the same movement periods (i.e., gait cycle) [38]. This time normalisation can result in a time shift of peak values due to variations in motion velocity. Hence, the results of SPM analysis – where data are compared at each time point – may differ from the results of discrete parameter analysis. Accordingly, the combination of time series analysis and time-independent discrete parameter analysis allows for a comprehensive analysis of leg differences and similarities in biomechanical and muscle activity parameters. Because weight-bearing and non-weight-bearing conditions affect secondary knee mechanics and knee contact forces differently [18, 39], we analysed the full gait cycle, stance and swing, during walking. Therefore, the trajectories and discrete parameters of knee kinematics and kinetics, and muscle activity were analysed and compared between the legs of ACL-IB (SSD = involved–uninvolved leg), between the involved legs of the patients (ACL-IB vs. ACL-R), and between the involved leg of patients and the non-dominant leg of the controls (ACL-IB vs. Controls, ACL-R vs. Controls).

*Trajectories.* Trajectories of knee kinematics and kinetics, and muscle activity data were compared using SPM (http://www.spm1D.org, M.0.4.7) [37]. Side-to-side differences in ACL-IB were detected using paired t-tests, and leg differences between groups were detected using a one-way ANOVA (P < 0.05) with post-hoc Bonferroni tests with a critical P value corrected for multiple comparisons (P < 0.05/3) [40]. Only significantly different intervals longer than 2%GC (i.e., from more than two consecutive time points) were analysed and interpreted [40, 41]. For these intervals (i.e., the difference between the trajectories exceeds the critical threshold), the mean and standard deviation (SD) of the maximum difference (mDiff) in each respective interval were calculated:

- SSD in ACL-IB: mean and SD of the maximum difference between the trajectory of the involved and uninvolved leg for each patient.
- Between ACL-IB and ACL-R: mean and SD of the maximum difference between the trajectory of each ACL-IB and the mean trajectory of ACL-R.
- Between patient groups (ACL-IB, ACL-R) and controls: mean and SD of the maximum difference between the trajectory of each patient and the mean trajectory of controls.

*Discrete parameters.* Frequently studied discrete parameters (maximum, minimum or mean) of knee angles, moments and power after ACL-R were calculated (Table 2) [2, 3, 33, 42]. For muscle activity data, the maximum activity of amplitude-normalized EMG data was calculated. 95% confidence intervals (CI) of SSD in ACL-IB and of leg differences between groups not containing zero were interpreted as significant leg differences (asymmetry). For these significant parameters, the mean and SD of the differences were also calculated.

## Results

### Spatio-temporal parameters

The groups did not differ in walking speed or in other spatio-temporal parameters (Table 2; Supplementary Table S1).

**Table 2:** Spatio-temporal parameters in patients after InternalBrace^TM^-augmented anterior cruciate ligament repair (ACL-IB), ACL reconstruction (ACL-R), and controls

*Parameter*	*ACL-IB* *(n = 29)*	*ACL-R* *(n = 27)*	*Controls* *(n = 29)*	*P value*
Walking speed (m/s)	1.38 (0.13)	1.41 (0.14)	1.43 (0.19)	0.443
Cadence (steps/min)	110 (5)	113 (8)	113 (8)	0.221
Stride time (s)	1.09 (0.05)	1.07 (0.08)	1.07 (0.08)	0.285
Stride length (m)	1.50 (0.13)	1.50 (0.11)	1.52 (0.16)	0.837

### Knee biomechanics

#### Side-to-side differences in ACL-IB

**Fig. 3:**
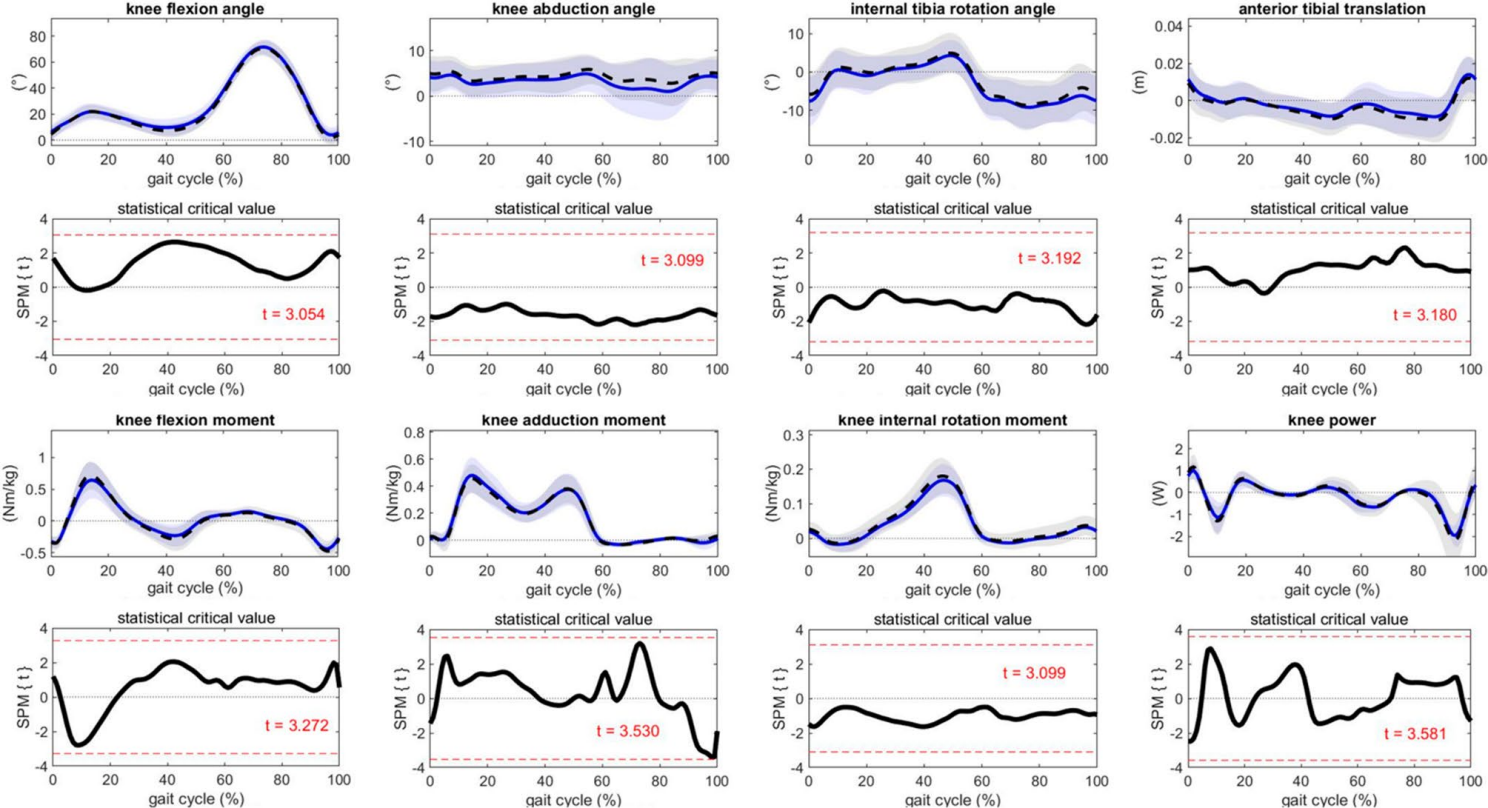
Mean (solid line) and 1 standard deviation (shaded area) of trajectories of knee biomechanics of the involved (blue) and uninvolved (dashed black) leg in patients after InternalBrace^TM^-augmented ACL-repair (ACL-IB), and statistical parametric mapping (SPM) analysis between the legs (t-test and statistical critical value (t))

*Trajectories.* No significant differences in knee biomechanics were found between the legs in ACL-IB (Fig. 3).

*Discrete parameters.* The minimal knee flexion angle in terminal stance (2.4 ± 5.1°) was significantly higher and the maximum knee flexion moment (-0.07 ± 0.17Nm/kg) was significantly lower in the involved leg than in the uninvolved leg in ACL-IB (CI excluding zero, Table 3).

#### Leg differences between groups

**Fig. 4:**
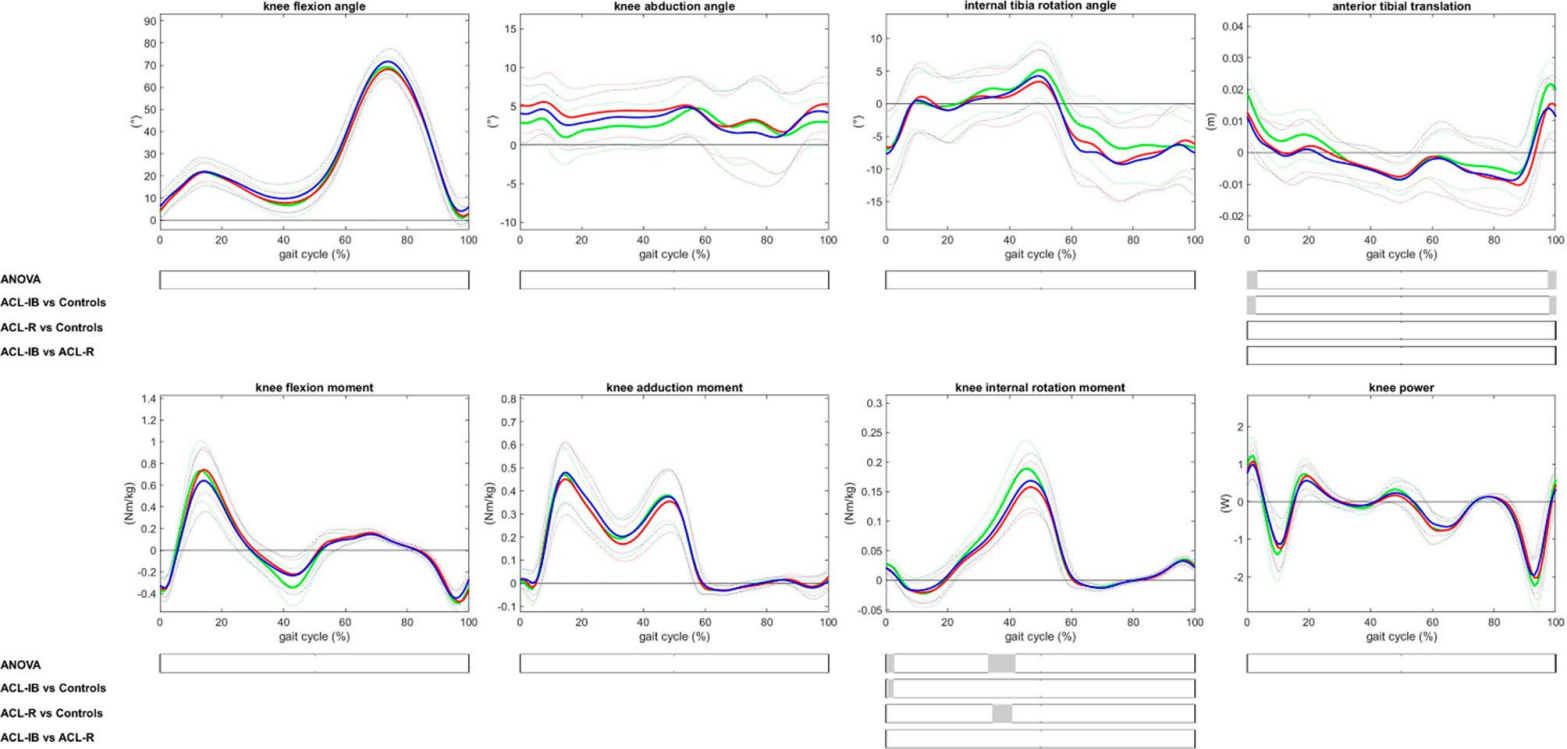
Mean (solid line) and 1 standard deviation (dashed line) of trajectories of knee biomechanics of the involved leg in patients after InternalBrace^TM^-augmented ACL repair (ACL-IB, blue), the involved leg in patients after ACL reconstruction (ACL-R, red) and the non-dominant leg of healthy controls (green), and intervals with significant difference (grey shaded area) revealed by statistical parametric mapping (one-way analysis of variance (ANOVA) with posthoc Bonferroni tests)

*Trajectories.* The trajectories of anterior tibia position (0–3%GC, P = 0.042; 97–100%GC, P = 0.045) and knee internal rotation moment (33–42%GC, P < 0.001; differences between 1–3%GC neglected) differed significantly between groups (Fig. 4). Post hoc analyses revealed differences only between patients and controls. ACL-IB had less anterior tibia position than controls before, at, and after foot strike (0–3%GC, P = 0.015, mDiff − 7.4 ± 9.2 mm; 98–100%GC, P = 0.016, mDiff − 8.9 ± 9.5 mm), and ACL-R had lower knee internal rotation moments than controls from 34 to 41%GC (P < 0.001, mDiff − 0.04 ± 0.03Nm/kg; difference ACL-IB vs. controls between 1–2%GC (neglected)).

*Discrete parameters.* During swing, ACL-IB knees were more maximally flexed than ACL-R knees (3.6 ± 7.6°, Table 3). Both ACL-IB (-6.2 ± 12.1 mm) and ACL-R (-5.9 ± 12.2 mm) had a lower maximum anterior tibia position during walking than controls. ACL-R also had significantly lower maximum knee extension moments (higher knee flexion moment, 0.13 ± 0.21Nm/kg) and lower maximum knee internal rotation moments than controls (-0.03 ± 0.07Nm/kg, Table 3).

### Muscle activity

#### Side-to-side differences in ACL-IB

**Fig. 5:**
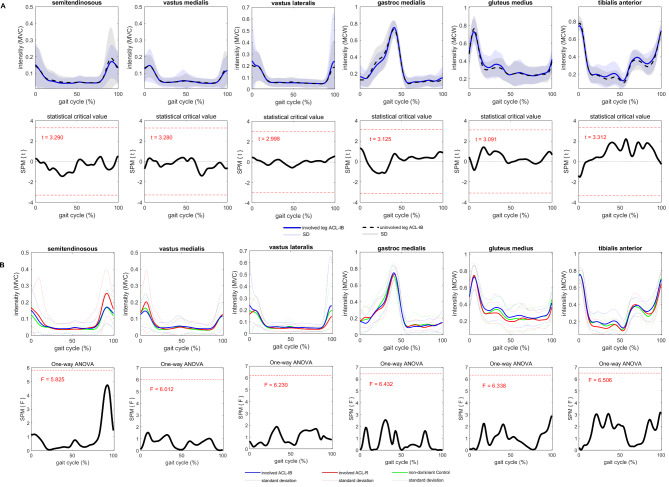
Mean (1 standard deviation) trajectories of lower leg muscle activity normalized to maximum voluntary isokinetic contraction (MVC, thigh muscles) or to maximum activation during walking (MCW, shank muscles). A: between legs in patients after InternalBrace^TM^-augmented ACL-repair (ACL-IB), and statistical parametric mapping analysis (SPM, t-test and statistical critical value (t)); B: between the involved leg in patients after ACL-IB, the involved leg in patients after ACL-R and the non-dominant leg of healthy controls, and SPM analysis (one-way analysis of variance (ANOVA)

*Trajectories and discrete parameters*. Muscle activation patterns did not differ between the involved and uninvolved leg in ACL-IB for either the SPM (Fig. 5A) or the discrete parameters (Table 3).

#### Leg differences between groups

*Trajectories*. No differences in muscle activity trajectories were found between groups (Fig. 5B).

*Discrete parameters*. In ACL-IB, none of the muscle activity parameters differed from ACL-R or from controls. ACL-R had a significantly higher maximum activity of the semitendinosus muscle during walking compared to controls (11.2 ± 25.3%MVC, Table 3).

**Table 3:** Discrete parameter of knee biomechanics and muscle activity in patients after InternalBraceTM-augmented anterior cruciate ligament repair (ACL-IB), ACL reconstruction (ACL-R) and controls

Parameter	*ACL-IB*		*ACL-R*	*Controls*	*Side-to-side difference*	*Leg difference between groups* *(Inv patients vs. NonDom controls)*		
	*Inv*	*UnInv*	*Inv*	*NoDom*	*ACL-IB*	*ACL-IB vs. Controls*	*ACL-R vs. Controls*	*ACL-IB vs. ACL-R*
	*mean (SD)*	*mean (SD)*	*mean (SD)*	*mean (SD)*	*95% CI*	*95% CI*	*95% CI*	*95% CI*
**Knee biomechanics**								
*Flexion angle (°)*								
Maximum, ST	22.4 (0.8)	22.3 (0.9)	21.9 (0.6)	22.0 (0.7)	[-2.1;2.2]	[-2.9;3.8]	[-3.5;3.3]	[-2.9;3.9]
Minimum, ST	9.3 (0.6)	6.9 (0.7)	7.5 (0.6)	6.4 (0.5)	**[0.5;4.4]**	[-0.7;6.5]	[-2.5;4.7]	[-1.8;5.4]
Maximum, SW	72.4 (0.5)	71.2 (0.4)	68.6 (0.9)	69.7 (0.6)	[-1.2;3.5]	[-0.4;5.9]	[-4.2;2.2]	**[0.5;7.0]**
*Abduction angle (°)*								
Mean, ST	3.2 (0.2)	4.2 (0.2)	4.0 (0.2)	2.6 (0.1)	[-2.1;0.0]	[-1.5;2.6]	[-0.8;3.5]	[-2.9;1.3]
*Internal rotation angle (°)*								
Mean, ST	-0.1 (0.5)	-0.8 (0.3)	-0.0 (0.4)	-0.8 (0.4)	[-0.9;2.3]	[-1.7;3.1]	[-1.6;3.2]	[-2.5;2.4]
Mean, SW	7.7 (0.5)	6.7 (0.8)	7.1 (0.5)	5.7 (0.3)	[-0.4;2.4]	[-0.9;4.9]	[-1.6;4.3]	[-2.3;3.6]
*Anterior tibia position (mm)*								
Maximum, GC	16.9 (0.9)	15.1 (1.2)	17.3 (1.0)	23.0 (0.7)	[-1.8;5.4]	**[-11.6;-0.7]**	**[-11.3;-0.2]**	[-6.0;5.1]
*Flexion moment (Nm/kg)*								
Maximum, ST	0.66 (0.04)	0.73 (0.05)	0.76 (0.03)	0.75 (0.03)	**[-0.13;-0.00]**	[-0.25;0.08]	[-0.17;0.17]	[-0.26;0.1]
Minimum, second half ST	-0.26 (0.02)	-0.31 (0.03)	-0.24 (0.02)	-0.36 (0.02)	[-0.00;0.11]	[-0.00;0.20]	**[0.02;0.23]**	[-0.13;0.1]
*Adduction moment (Nm/kg)*								
First maximum, ST	0.51 (0.03)	0.48 (0.03)	0.47 (0.02)	0.49 (0.02)	[-0.01;0.06]	[-0.07;0.10]	[-0.10;0.07]	[-0.05;0.1]
Second maximum ST	0.39 (0.02)	0.39 (0.02)	0.36 (0.02)	0.40 (0.02)	[-0.04;0.03]	[-0.08;0.07]	[-0.11;0.05]	[-0.05;0.1]
*Internal rotation moment (Nm/kg)*								
Maximum, ST	0.17 (0.01)	0.18 (0.01)	0.16 (0.01)	0.19 (0.01)	[-0.03;0.01]	[-0.05;0.01]	**[-0.06;-0.00]**	[-0.02;0.0]
*Power (W/kg)*								
Maximum, ST	1.11 (0.09)	1.25 (0.12)	1.17 (0.05)	1.36 (0.08)	[-0.32;0.03]	[-0.55;0.05]	[-0.49;0.12]	[-0.37;0.2]
Minimum, ST	-1.28 (0.14)	-1.43 (0.19)	-1.38 (0.09)	-1.51 (0.12)	[-0.00;0.29]	[-0.18;0.64]	[-0.29;0.55]	[-0.32;0.5]
Minimum, SW	-2.22 (0.09)	-2.26 (0.08)	-2.24 (0.06)	-2.45 (0.14)	[-0.09;0.14]	[-0.16;0.62]	[-0.19;0.59]	[-0.37;0.4]
**Muscle activity**								
*Maximum activation during gait cycle*								
Semitendinosus (%MVC)	22.0 (2.6)	22.9 (2.7)	30.7 (4.3)	20.0 (2.7)	[-7.6;5.9]	[-8.3;12.3]	**[0.1;21.3]**	[-19.2;1.8]
Vastus medialis (%MVC)	17.4 (3.2)	17.7 (2.7)	22.4 (8.5)	18.4 (2.6)	[-3.8;3.3]	[-10.6;8.7]	[-5.9;13.8]	[-14.8;4.9]
Vastus lateralis (%MVC)	32.1 (13.0)	25.9 (9.7)	23.0 (5.8)	25.6 (5.7)	[-4.3;17.6]	[-13.7;26.7]	[-22.8;17.7]	[-11.5;29.6]
Gastrocnemius medialis (%MCW)	81.7 (3.8)	80.4 (7.4)	80.4 (3.9)	81.3 (3.1)	[-2.7;6.9]	[-3.7;4.6]	[-5.3;3.5]	[-3.1;5.8]
Gluteus medius (%MCW)	82.5 (3.2)	82.1 (2.8)	83.8 (4.1)	82.4 (4.0)	[-3.7;3.9]	[-4.2;4.6]	[-3.0;5.9]	[-5.8;3.2]
Tibialis anterior (%MCW)	83.2 (2.8)	85.8 (3.8)	83.4 (4.0)	84.2 (2.9)	[-5.9;0.9]	[-4.6;2.6]	[-4.5;2.8]	[-3.8;3.5]

Inv, involved leg; UnInv, Uninvolved leg; NonDom, non-dominant leg; SD, standard deviation; CI, confidence Interval; GC: gait cycle; ST, stance phase; SW, swing phase; MVC, isokinetic maximum voluntary contraction; MCW, maximum contraction during walking

Bold printed values indicate that 95%CI is excluding zero, indicating a significant difference

## Discussion

Our aim was to determine differences in knee kinematics and kinetics, and muscle activity during walking between the legs of patients 2 years after ACL-IB (SSD), and to compare the same parameters between the involved legs of patients 2 years after ACL-IB, the involved legs of patients 2 years after ACL-R, and the non-dominant leg of healthy controls. Our results confirmed our first hypothesis that there would be no SSDs in patients 2 years after ACL-IB in almost all parameters. Our second hypothesis, that knee biomechanics and muscle activation patterns would be more natural (closer to control) after ACL-IB than after ACL-R was partially confirmed, particularly for knee moments and semitendinosus muscle activity.

### Side-to-side differences in ACL-IB

During loading response, patients after ACL-IB had a lower maximum knee flexion moment in the involved leg compared to the uninvolved leg, but not compared to the non-dominant leg in our control group. During push-off, in ACL-IB, the involved knee was 2.4° more flexed, resulting in a lower range of motion during the stance phase of walking. However, regardless of statistical significance, this difference was less than the minimal clinically important difference of 3° reported in the literature for differences between limbs in a healthy population [43], and less than 4°, the error reported in a systematic review of the reliability of kinematics in 3D gait analysis for the sagittal plane [44].

During loading response, patients after ACL-IB had a lower maximum knee flexion moment in the involved leg compared to the uninvolved leg, but not compared to the non-dominant leg in our control group. The external knee flexion moment is internally controlled by the quadriceps muscle, whose contraction is associated with anterior tibial shear force and tibia translation [45]. The ACL is considered to be the main constraint against anterior tibial translation [46]. Consequently, the observed lower maximum knee flexion moment in patients after ACL-IB could be a mechanism that probably relieves or protects the healed ACL or the involved knee. Nevertheless, the 95% CIs of the SSD in ACL-IB overlapped with the CIs of our age- and sex-matched healthy control group for this parameter (Supplements Table S1), suggesting that these differences are not greater than those observed in the controls. Furthermore, the interlimb difference in knee flexion moment was at the margin of 0.04 Nm/kg*m (0.07 Nm/kg ≈ 0.04 Nm/kg*m) and thus within the minimal clinically important difference reported by Di Stasi et al. [43]. The average leg asymmetry in the maximum external knee flexion moment in ACL-IB was approximately 9.6%. An asymmetry of 10% in this parameter during walking was assumed to be exceeded by 72–84% of the total population, questioning the generally considered threshold of 10% asymmetry for joint kinetics [47] and the relevance of asymmetry in ACL-IB.

Based on these results and the thresholds reported in the literature, we consider the observed interlimb differences in minimum knee extension angle during push-off and in maximum knee flexion moment during loading response in ACL-IB to be not meaningful, suggesting that there is no relevant asymmetry in knee kinematics, kinetics, and muscle activity 2 years after ACL-IB. Nevertheless, these differences should be considered in the context of possible subsequent injury or the onset of osteoarthritis and may continue to decrease, stagnate, or even increase over time.

### Differences between groups

#### Knee biomechanics

The greater knee flexion angle during swing in ACL-IB compared to ACL-R was the only significant difference observed between patient groups. As mentioned previously, the observed difference of < 4° in knee flexion angle was rather small and within the error for knee flexion angles recorded using skin-based motion capture [44], suggesting that there is no meaningful difference in knee biomechanics, and in muscle activation between ACL-IB and ACL-R 2 years after surgery. This result is consistent with the findings of Schlieman et al. [48] who found no differences in knee flexion angle between ACL-R and a dynamic augmented ACL repair technique at 6 weeks and 6 months postoperatively.

The comparison between ACL patients and the matched healthy control group with comparable knee-related activity levels shown in the TAS indicated similar gait adaptation in knee biomechanics after ACL-IB and ACL-R, especially in anterior tibia position and knee moments. Although only patients after ACL-IB showed a lower anterior tibia position around foot strike compared with controls when comparing trajectories (SPM), a significant difference was also observed between ACL-R and controls in maximum anterior tibia position (discrete parameter). Therefore, the translational behaviour of the tibia does not seem to be fully recovered even 2 years after ACL-IB or ACL-R. As stated previously, differences in the results between the SPM analysis and the discrete parameter analysis may occur due to variations in motion velocity affected by time normalisation. Therefore, peak values may be shifted (rather than coinciding in time), which may have resulted in non-significant (masked) differences in the SPM analysis between ACL-R and controls when comparing each time point between groups.

Inconsistent results have been reported for dynamic anterior-posterior translation in the knee during walking after ACL-R, mostly comparing between legs in patient and not to controls. Beard et al. [49] observed greater relative anterior tibia translation in the involved compared with the uninvolved leg throughout the gait cycle at 6 months after ACL-R. Erhart-Hledik et al. [33] reported an average anterior position of the femur relative to the tibia (posterior tibia position relative to the femur) during stance in the involved leg 2 years after ACL-R, whereas the uninvolved leg had an average posterior position of the femur, suggesting a reduction in anterior tibia translation. Tagesson et al. [42] observed a greater range of anterior tibia translation during the stance phase of walking in the involved leg compared to the uninvolved leg 5 years after ACL-R (hamstring tendon autografts). We only observed differences in anterior tibia position in the involved leg of our patients compared to controls but not between the legs of ACL-IB (and ACL-R, Supplements Table S1), in contrast to results reported in the literature [33, 42, 49]. The differences between our data and these studies may be due to different measurement techniques (electrogoniometer [42], marker-based [33, 49]), calculation of translation (inverse kinematics [33], relative marker displacements [49]), and/or different reference positions (passively guided knee motion [42], standing position [33]). In general, marker-based analyses of kinematics, especially joint displacements, are affected by soft tissue artefacts [50, 51]. Therefore, results on absolute tibia-femur position should be interpreted with caution.

While lower maximum knee extension (higher knee flexion) moment and lower (maximum) internal rotation moments were observed in ACL-R compared to controls in SPM and/or discrete parameters, ACL-IB did not differ significantly from controls in these parameters. Our results after ACL-R are comparable to two meta-analyses [2, 3] that still showed deviations in knee moments in patients after ACL-R. Slater et al. [3] found that maximum external moments in knee flexion, extension, adduction, and external rotation were lower in ACL-R than in controls. Kaur et al. [2] reported lower maximum knee flexion moments (strong evidence) and first maximum knee adduction moment (moderate to strong evidence) in ACL-R compared to controls. Our results are partially consistent with these findings, but only for lower maximum knee extension (minimum knee flexion) and internal rotation moments during the second half of stance after ACL-R. While the absolute differences between ACL-R and controls in maximum knee extension (minimum knee flexion) moment were above the minimum clinically important difference reported by Di Stasi et al. (-0.13 Nm/kg > 0.04 Nm/kg*m ≈ 0.07 Nm/kg) [43], the difference in internal tibia rotation moment was not (-0.03 Nm/kg). However, this threshold was originally specified for knee flexion moments only. Nevertheless, we also observed a non-significant trend towards lower maximum knee extension (higher knee flexion) and maximum internal rotation moments in ACL-IB compared to controls. The CIs of the difference between ACL-IB and controls in these parameters barely included zero. Furthermore, the patients after ACL-IB did not differ significantly from ACL-R in these parameters and had a similar mean difference to controls as ACL-R (for comparison: ACL-IB, mean maximum knee extension (maximum knee flexion) moment: 0.10 Nm/kg; mean maximum knee internal rotation moment -0.02 Nm/kg). Therefore, our results support a comparable gait adaptation strategy in terms of knee kinetics after ACL-IB and ACL-R, which is further strengthened by our complementary analyses of hip joint kinetics in the supplements. Nevertheless, the differences in knee kinetics seem to be slightly (non-significantly) smaller in ACL-IB than in ACL-R compared to controls, and it remains to be investigated whether this trend is more pronounced (or even different between the two ACL groups) in highly dynamic tasks such as running or jumping.

Interestingly, most of the differences in the involved leg of ACL patients were observed around foot strike and in terminal stance around ipsilateral push-off, compared to the healthy contralateral leg or with controls. During a complete gait cycle, the maximum length of the ACL or its posteromedial/anterolateral bundle was measured immediately before, at, or shortly after foot strike [52–55], during midstance [54, 55], and during the push-off phase [52, 53, 56], i.e., at ipsilateral heel rise. A positive relationship between knee extension and length of the ACL has also been reported [52, 53, 55]. Given these findings, it is not surprising that most of the differences in our two ACL groups were found around foot strike and push-off of the involved leg when the knee is close to extension. Therefore, the observed differences or trends in both groups compared to controls may be a mechanism to unload or (preemptively) protect the healed or reconstructed ACL from loading or stretching.

#### Muscle activity

During walking, patients after ACL-R used up to 11% more of their maximal voluntary semitendinosus activation than controls (for comparison: mean ACL-IB vs. controls: +2.1%; mean ACL-IB vs. ACL-R: -8.7%). The tendency for higher semitendinosus activation in ACL-R compared to controls was also observed in SPM, although not significantly. This could be due to the discrepancy between SPM and discrete parameters (discussed in 4.2.1) or the high variability in semitendinosus activity in ACL-R compared to controls and ACL-IB. A previous meta-analysis [4] showed moderate to strong evidence of higher hamstring EMG amplitude during walking and stair climbing after ACL-R (55% hamstring tendon grafts) compared to controls when EMG signals were normalized to maximum voluntary isometric contraction. Our results observed after ACL-R are consistent with these findings and are also supported by complementary results of higher semitendinosus muscle activity in the involved leg than the uninvolved leg after ACL-R (Supplements, Table S1, Figure S5A). It is possible that patients after ACL-R have a lower maximal voluntary contraction after semitendinosus harvesting, and consequently have to use more of their muscle activation capacity during walking in order to maintain ambulation. Because ACL repair does not require tendon harvesting and thus does not involve additional intervention in the muscle-tendon complex, it is not surprising that we did not observe altered activation patterns after ACL-IB. However, we did not observe significant differences between the two groups of patients, which may be due to a slightly underpowered ACL-R group. The impairments in semitendinosus muscle function [4, 57–59] and proprioception [60] reported after ACL-R may explain the observed differences compared to controls. However, the presumed preservation of proprioception after ACL-IB remains unclear.

The loading of the knee joint or ACL depends not only on the magnitude and direction of the external forces, i.e., ground reaction force, but also on the magnitude and direction of the internal forces, such as the forces exerted by the muscles spanning the knee [20], and therefore the knee muscles can load or unload the ACL [45]. The hamstring muscles are considered agonists of the ACL. When contracted, they prevent forward displacement of the tibia, thereby unloading the ACL, especially in more flexed knee positions [19]. In addition, hamstring muscle activity has been shown to influence the magnitude and timing of ACL loading during single-leg landing [21]. Therefore, the higher activity of the semitendinosus muscle in patients after ACL-R (which occurs predominantly around heel strike) may also stabilize the knee in a preparatory and protective manner, limiting anterior tibia translation and anterior shear forces during foot strike and loading response. This hypothesis is supported by previously published studies [61–64]. In ACL-deficient patients, greater medial and lateral hamstring muscle activity, and higher hamstrings-to-quadriceps co-contraction of the medial and lateral pairs were observed in the involved leg compared to the healthy contralateral leg during weight-acceptance of walking [61]. After ACL-R (32% hamstring tendon grafts), higher co-activation of medial and lateral hamstrings and quadriceps, as well as semitendinosus and vastus medialis was reported compared to controls during the foot strike phase of walking [62]. Recently, in healthy subjects with lax knees, higher activity of the semitendinosus muscle was observed when anterior tibia translation was higher during jump landings [63]. Thus, hamstring muscle activity appears to be a natural mechanism for limiting anterior translation. Furthermore, higher semitendinosus muscle activity before foot strike during jump landing was found in patients after primary ACL-R compared to patients after secondary ACL-R (all patellar tendon grafts) [64]. Therefore, higher activity was considered protective for secondary ACL rupture after ACL-R. Whether such a protective mechanism also exists after ACL-IB remains to be clarified. However, it could be that after ACL-IB a higher activity of the semitendinosus is not necessary for compensation or prevention because the semitendinosus was not harvested.

In conclusion, the similarity in muscle activity after ACL-IB compared to controls but the presence of differences after ACL-R, suggests a reduced or even absent muscular compensatory mechanism after ACL-IB. Consequently, our results highlight that preservation of native ACL fibres and the semitendinosus muscle including its tendon may be beneficial.

### Clinical implications

These results indicate that neither ACL group was functionally worse or better, as demonstrated by comparable ambulatory knee mechanics, and muscle activity patterns in a direct comparison between ACL-IB and ACL-R. Regarding the surgical procedure, ACL-IB achieved a similar walking outcome while being less invasive and preserving the surrounding knee musculature, including its function. Thus, the absence of harvesting and associated complications [4] with ACL repair seems to be an advantage over ACL-R. Therefore, our results demonstrate that there is the potential for InternalBrace^TM^-augmented ACL repair as an alternative treatment modality for a specific subgroup of ACL patients (i.e., adult patients, proximal ACL rupture, operated within 3 weeks of ACL injury, moderate knee-related activity level). However, it is not known whether these results hold true when compared to patients with other tendon grafts. Regardless of ACL-IB or ACL-R, neither group achieved normal walking biomechanics (translation and kinetics). Therefore, 2 years postoperatively, gait still appears to be affected by the original ACL rupture and may not fully normalize thereafter. This is consistent with the notion that ACL rupture not only represents a mechanical disruption in terms of loss of stability, but also affects sensorimotor integrity, including neuroplastic adaptations [65]. Therefore, neurological, sensorimotor studies are needed in addition to biomechanical analyses to understand the full functional outcomes after augmented ACL repair and the potential differences/benefits of this surgical technique compared to reconstruction.

### Strength and limitations

The major strength of this study is that, for the first time, we provide information on knee biomechanics and leg muscle activity patterns after ACL-IB and compare them with age- and sex-matched patients after ACL-R with hamstring tendon grafts and a healthy matched control group. This study also has some limitations. Based on surgical recommendations, we only included patients with a proximal rupture for augmented ACL repair [8], whereas all rupture lesions were accepted for ACL-R. Therefore, our results may be biased by the selection of ACL patients. Both groups of patients underwent standard physical therapy, but the duration and adherence to therapy were not recorded, which may have further influenced our results. We informed our subjects to bring comfortable (sports) shoes, as the test battery of the umbrella study [22] included other dynamic activities. The extent to which different shoe types may have influenced our results is unknown. Because we used two different normalisation methods, the interpretation of the thigh muscles in combination with the other muscles is limited. Finally, angular rotations and especially joint translations [47] measured using skin marker motion analysis are highly dependent on marker placement and subject to soft tissue artefacts [61–63]. Therefore, the absolute values and the magnitude of the differences observed (e.g., especially the values for anterior tibia position) may be higher than usual [47]. However, as all participants were analysed using the same method, it can be assumed that all participants are subject to approximately the same systematic errors, so the observed differences are unlikely to have occurred by chance.

## Conclusion

Two years after ACL-IB, patients have no relevant leg asymmetry in knee biomechanics or muscle activity. The similar walking outcome after ACL-IB and ACL-R, but the less invasive surgery of ACL-IB (i.e., no tendon harvest), underlines the value of augmented ACL repair as a possible alternative to ACL-R for patients with a proximal rupture and a moderate activity level (median, TAS of 4). Nevertheless, both groups of patients still showed gait adaptations compared to controls at 2 years postoperatively. Fewer differences in knee kinetics and semitendinosus muscle activation after ACL-IB than after ACL-R indicates that preservation of native ACL fibres and the muscle-tendon complex may be beneficial. Biomechanical and neurological sensorimotor studies are desirable to better understand the differences in functional outcome between ACL-IB and ACL-R and the presumed benefits of ACL-IB.

### Electronic supplementary material

Below is the link to the electronic supplementary material.

Supplementary Material 1

## Data Availability

All data generated or analysed during this study are included in this published article and its supplementary information files (Table S1: Spatio-temporal and discrete parameter analysis within and between all groups; Figure S1-3: Trajectories of knee biomechanics and ground reaction force of both legs in ACL-IB, ACL-R and controls; Figure S4: Trajectories of hip and ankle biomechanics of the involved leg of patients compared to the non-dominant of controls; Figure S5–A/B: Trajectories of muscle activity of both legs in ACL-R and controls). Source data are available by reasonable request to the corresponding author.

## Abbreviations

ACL: anterior cruciate ligament
ANOVA: analysis of variance
CGM: conventional gait model
CI: confidence interval
EMG: electromyography
GC: gait cycle
IB: InternalBrace™ (Arthrex Inc.)
IC: initial contact
MVC: maximum voluntary contraction
PCT: point cluster technique
R: reconstruction
SSD: side-to-side difference
TAS: Tegner activity score

## References

[1] Filbay SR, Grindem H (2019). Evidence-based recommendations for the management of anterior cruciate ligament (ACL) rupture. Best Pract Res Clin Rheumatol.

[2] Kaur M, Ribeiro DC, Theis J-C, Webster KE, Sole G (2016). Movement patterns of the knee during Gait following ACL Reconstruction: a systematic review and Meta-analysis. Sports Med.

[3] Slater LV, Hart JM, Kelly AR, Kuenze CM (2017). Progressive changes in walking kinematics and Kinetics after Anterior Cruciate Ligament Injury and Reconstruction: a review and Meta-analysis. J Athl Train.

[4] Sherman DA, Glaviano NR, Norte GE (2021). Hamstrings neuromuscular function after Anterior Cruciate Ligament Reconstruction: a systematic review and Meta-analysis. Sports Med.

[5] Davis K, Williams JL, Sanford BA, Zucker-Levin A (2019). Assessing lower extremity coordination and coordination variability in individuals with anterior cruciate ligament reconstruction during walking. Gait Posture.

[6] Cinque ME, Dornan GJ, Chahla J, Moatshe G, LaPrade RF (2018). High rates of Osteoarthritis develop after anterior cruciate ligament surgery: an analysis of 4108 patients. Am J Sports Med.

[7] Li J, Rothrauff B, Chen S, Zhao S, Wu Z, Chen Q, He J (2022). Trends in Anterior Cruciate Ligament Repair: a bibliometric and visualized analysis. Orthop J Sports Med.

[8] van der List JP, DiFelice GS (2017). Arthroscopic primary anterior cruciate ligament repair with suture augmentation. Arthrosc Tech.

[9] Richmond JC. Anterior Cruciate Ligament Reconstruction. Sports Med Arthrosc Rev. 2018:165–7. 10.1097/JSA.0000000000000218 30395059

[10] Murray MM, Fleming BC (2013). Use of a bioactive scaffold to stimulate anterior cruciate ligament healing also minimizes posttraumatic osteoarthritis after surgery. Am J Sports Med.

[11] Rilk S, Goodhart GC, O’Brien R, Vermeijden HD, van der List JP, DiFelice GS (2023). Anatomic arthroscopic primary repair of proximal anterior cruciate ligament tears. Arthrosc Tech.

[12] Chahla J, Nelson T, Dallo I, Yalamanchili D, Eberlein S, Limpisvasti O (2020). Anterior cruciate ligament repair versus reconstruction: a kinematic analysis. Knee.

[13] Leister I, Kulnik ST, Kindermann H, Ortmaier R, Barthofer J, Vasvary I (2019). Functional performance testing and return to sport criteria in patients after anterior cruciate ligament injury 12–18 months after index surgery: a cross-sectional observational study. Phys Ther Sport.

[14] Ferreira A, Saithna A, Carrozzo A, Guy S, Vieira TD, Barth J, Sonnery-Cottet B (2022). The minimal clinically important difference, patient Acceptable Symptom State, and clinical outcomes of Anterior Cruciate Ligament Repair Versus Reconstruction: a matched-pair analysis from the SANTI Study Group. Am J Sports Med.

[15] Mattiassich G, Ortmaier R, Kindermann H, Barthofer J, Vasvary I, Kulnik ST (2020). Klinische und radiologische Ergebnisse nach Naht des vorderen Kreuzbandes mittels Internal-Brace- und all-inside-Kreuzbandersatzplastik nach 12–18 Monaten nach Operation. [Clinical and radiological results after Internal Brace suture versus the all-inside reconstruction technique in anterior cruciate ligament tears 12 to 18 months after index surgery]. Sportverletz Sportschaden.

[16] Szwedowski D, Paczesny Ł, Zabrzyński J, Gagat M, Domżalski M, Huri G, Widuchowski W (2021). The comparison of clinical result between primary repair of the anterior cruciate ligament with additional internal bracing and anatomic single Bundle Reconstruction-A Retrospective Study. J Clin Med.

[17] Pang L, Li P, Li T, Li Y, Zhu J, Tang X (2022). Arthroscopic anterior cruciate ligament repair Versus Autograft Anterior Cruciate Ligament Reconstruction: a Meta-analysis of comparative studies. Front Surg.

[18] Thomeer L, Guan S, Gray H, Schache A, de Steiger R, Pandy M (2020). Six-degree-of-freedom Tibiofemoral and Patellofemoral Joint Motion during Activities of Daily Living. Ann Biomed Eng.

[19] Imran A, O’Connor JJ (1998). Control of knee stability after ACL injury or repair: interaction between hamstrings contraction and tibial translation. Clin Biomech Elsevier Ltd.

[20] Shelburne KB, Pandy MG, Anderson FC, Torry MR (2004). Pattern of anterior cruciate ligament force in normal walking. J Biomech.

[21] Ueno R, Navacchia A, Schilaty ND, Myer GD, Hewett TE, Bates NA (2021). Hamstrings Contraction regulates the magnitude and timing of the peak ACL loading during the Drop Vertical Jump in female athletes. Orthop J Sports Med.

[22] Müller S, Bühl L, Nüesch C, Pagenstert G, Mündermann A, Egloff C (2022). RetroBRACE: clinical, socioeconomic and functional-biomechanical outcomes 2 years after ACL repair and InternalBrace augmentation in comparison to ACL reconstruction and healthy controls-experimental protocol of a non-randomised single-centre comparative study. BMJ Open.

[23] Shermann MF, Lieber L, Bonamo JR, Podesta L, Reiter I. The long-term followup of primary anterior cruciate ligament repair: defining a rationale for augmentation. Am J Sports Med. 1991:243. 10.1177/036354659101900307 1867333

[24] van der List JP, DiFelice GS. Preservation of the anterior cruciate ligament: a treatment algorithm based on tear location and tissue quality. Am J Orthop. 2016;45(7):E393–E405. 28005092

[25] Bühl L, Müller S, Nüesch C, Pagenstert G, Mündermann A, Egloff C. Functional performance 2 years after ACL surgery: InternalBraceTM-augmented repair versus reconstruction versus healthy controls. J Orthop Traumatol. 2023;24:52. 10.1186/s10195-023-00723-5. 10.1186/s10195-023-00723-5 PMC10513977 37735271

[26] Müller S, Bühl L, Nüesch C, Pagenstert G, Mündermann A, Egloff C. Favorable patient reported, clinical and functional outcomes 2 years after ACL repair and InternalBraceTM augmentation compared with ACL reconstruction and healthy controls. Am J Sports Med. 2023:3635465231194784. 10.1177/03635465231194784. 10.1177/03635465231194784 PMC10543955 37675973

[27] Tegner Y, Lysholm J (1985). Rating Systems in the evaluation of knee ligament injuries. Clin Orthop Relat Res.

[28] Harris PA, Taylor R, Minor BL, Elliott V, Fernandez M, O’Neal L (2019). The REDCap consortium: building an international community of software platform partners. J Biomed Inform.

[29] Harris PA, Taylor R, Thielke R, Payne J, Gonzalez N, Conde JG (2009). Research electronic data capture (REDCap)- -a metadata-driven methodology and workflow process for providing translational research informatics support. J Biomed Inform.

[30] Woltring HJ (1986). A Fortran package for generalized, cross-validatory spline smoothing and differentiation. Adv Eng Softw.

[31] Dyrby CO, Andriacchi TP (2004). Secondary motions of the knee during weight bearing and non-weight bearing activities. J Orthop Res.

[32] Hafer JF, Kent JA, Boyer KA (2019). Physical activity and age-related biomechanical risk factors for knee osteoarthritis. Gait Posture.

[33] Erhart-Hledik JC, Chu CR, Asay JL, Andriacchi TP (2018). Longitudinal changes in knee gait mechanics between 2 and 8 years after anterior cruciate ligament reconstruction. J Orthop Res.

[34] Leboeuf F, Baker R, Barré A, Reay J, Jones R, Sangeux M (2019). The conventional gait model, an open-source implementation that reproduces the past but prepares for the future. Gait Posture.

[35] Hermens HJ (1999). European recommendations for surface ElectroMyoGraphy: results of the SENIAM project.

[36] Winter DA (2009). Biomechanics and motor control of human movement.

[37] Pataky TC, Robinson MA, Vanrenterghem J (2016). Region-of-interest analyses of one-dimensional biomechanical trajectories: bridging 0D and 1D theory, augmenting statistical power. PeerJ.

[38] Pataky TC, Robinson MA, Vanrenterghem J (2013). Vector field statistical analysis of kinematic and force trajectories. J Biomech.

[39] Johal P, Williams A, Wragg P, Hunt D, Gedroyc W (2005). Tibio-femoral movement in the living knee. A study of weight bearing and non-weight bearing knee kinematics using ‘interventional’ MRI. J Biomech.

[40] Wesseling M, Meyer C, Corten K, Desloovere K, Jonkers I (2018). Longitudinal joint loading in patients before and up to one year after unilateral total hip arthroplasty. Gait Posture.

[41] de Pieri E, Nüesch C, Pagenstert G, Viehweger E, Egloff C, Mündermann A (2022). High tibial osteotomy effectively redistributes compressive knee loads during walking. J Orthop Res.

[42] Tagesson S, Öberg B, Kvist J (2015). Static and dynamic tibial translation before, 5 weeks after, and 5 years after anterior cruciate ligament reconstruction. Knee Surg Sports Traumatol Arthrosc.

[43] Di Stasi SL, Snyder-Mackler L (2012). The effects of neuromuscular training on the gait patterns of ACL-deficient men and women. Clin Biomech (Bristol Avon).

[44] McGinley JL, Baker R, Wolfe R, Morris ME (2009). The reliability of three-dimensional kinematic gait measurements: a systematic review. Gait Posture.

[45] Maniar N, Cole MH, Bryant AL, Opar DA (2022). Muscle force contributions to Anterior Cruciate Ligament Loading. Sports Med.

[46] Butler RJ, Noyes NR, Grood ES (1980). Ligamentous restraints to anterior drawer in the human knee: a biomechanical study. JBJS.

[47] Lathrop-Lambach RL, Asay JL, Jamison ST, Pan X, Schmitt LC, Blazek K (2014). Evidence for joint moment asymmetry in healthy populations during gait. Gait Posture.

[48] Schliemann B, Glasbrenner J, Rosenbaum D, Lammers K, Herbort M, Domnick C (2018). Changes in gait pattern and early functional results after ACL repair are comparable to those of ACL reconstruction. Knee Surg Sports Traumatol Arthrosc.

[49] Beard DJ, Murray DW, Gill HS, Price AJ, Rees JL, Alfaro-Adrián J, Dodd CAF (2001). Reconstruction does not reduce tibial translation in the cruciate-deficient knee. J Bone Joint Surg Br.

[50] Benoit DL, Ramsey DK, Lamontagne M, Xu L, Wretenberg P, Renström P (2006). Effect of skin movement artifact on knee kinematics during gait and cutting motions measured in vivo. Gait Posture.

[51] Benoit DL, Ramsey DK, Lamontagne M, Xu L, Wretenberg P, Renström P (2007). In vivo knee kinematics during gait reveals new rotation profiles and smaller translations. Clin Orthop Relat Res.

[52] Englander ZA, Wittstein JR, Goode AP, Garrett WE, Defrate LE (2020). Reconsidering reciprocal length patterns of the Anteromedial and Posterolateral Bundles of the Anterior Cruciate Ligament during in vivo gait. Am J Sports Med.

[53] Nagai K, Gale T, Chiba D, Su F, Fu F, Anderst W. The Complex Relationship Between In Vivo ACL Elongation and Knee Kinematics During Walking and Running. J Orthop Res. 2019;37:1920–8. 10.1002/jor.24330. 10.1002/jor.24330 PMC6719793 31042309

[54] Wu JL, Hosseini A, Kozanek M, Gadikota HR, Gill TJ, Li G. Kinematics of the anterior cruciate ligament during gait. Am J Sports Med. 2010;38:1475–1482. 10.1177/0363546510364240. 10.1177/0363546510364240 PMC3740375 20442323

[55] Taylor KA, Cutcliffe HC, Queen RM, Utturkar GM, Spritzer CE, Garrett WE, DeFrate LE (2013). In vivo measurement of ACL length and relative strain during walking. J Biomech.

[56] Roldán E, Reeves ND, Cooper G, Andrews K (2017). In vivo mechanical behaviour of the anterior cruciate ligament: a study of six daily and high impact activities. Gait Posture.

[57] Bryant AL, Clark RA, Pua Y-H (2011). Morphology of hamstring torque-time curves following ACL injury and reconstruction: mechanisms and implications. J Orthop Res.

[58] Rush JL, Norte GE, Lepley AS (2020). Limb differences in hamstring muscle function and morphology after anterior cruciate ligament reconstruction. Phys Ther Sport.

[59] Ristanis S, Tsepis E, Giotis D, Stergiou N, Cerulli G, Georgoulis AD (2009). Electromechanical delay of the knee flexor muscles is impaired after harvesting hamstring tendons for anterior cruciate ligament reconstruction. Am J Sports Med.

[60] Busch A, Blasimann A, Mayer F, Baur H (2021). Alterations in sensorimotor function after ACL reconstruction during active joint position sense testing. A systematic review. PLoS ONE.

[61] Hurd WJ, Snyder-Mackler L (2007). Knee instability after acute ACL rupture affects movement patterns during the mid-stance phase of gait. J Orthop Res.

[62] Blackburn T, Pietrosimone B, Goodwin JS, Johnston C, Spang JT (2019). Co-activation during gait following anterior cruciate ligament reconstruction. Clin Biomech (Bristol Avon).

[63] Keizer MNJ, Hijmans JM, Gokeler A, Benjaminse A, Otten E (2020). Healthy subjects with lax knees use less knee flexion rather than muscle control to limit anterior tibia translation during landing. J Exp Orthop.

[64] Palmieri-Smith RM, Strickland M, Lepley LK (2019). Hamstring muscle activity after primary Anterior Cruciate Ligament Reconstruction-A Protective mechanism in those who do not sustain a secondary Injury? A preliminary study. Sports Health.

[65] Grooms DR, Page SJ, Nichols-Larsen DS, Chaudhari AMW, White SE, Onate JA (2017). Neuroplasticity Associated with Anterior Cruciate Ligament Reconstruction. J Orthop Sports Phys Ther.

