# Supplementary material for: Ambulatory knee biomechanics and muscle activity 2 years after ACL surgery: InternalBraceTM-augmented ACL repair versus ACL reconstruction versus healthy controls
Source: BMC Musculoskelet Disord. 2023 Oct 4;24:785. doi: 10.1186/s12891-023-06916-7 (PMC10548591; doi:10.1186/s12891-023-06916-7)
Supplement: Supplementary file 1 — Supplementary Material 1 [file 12891_2023_6916_MOESM1_ESM.pdf]

Supplement Table S1: Mean (1 standard deviation) of spatio-temporal and discrete parameter (maxima, minima or mean during gait cycle (GC, 0-100%GC), stance (ST, 0-60%GC) or swing (SW, 61-100%GC)) of kinematics and kinetics in hip, knee and ankle joint, ground reaction force (GRF) and muscle activity of semitendinosus, vastus medialis, vastus lateralis, gastrocnemius medialis, gluteus medius and tibialis anterior muscle in patients after InternalBrace^{TM}-augmented ACL repair (ACL-IB), ACL-reconstruction (ACL-R) and in healthy controls. 95% confidence intervals (CI) of leg difference within groups (involved – uninvolved; non-dominant – dominant) and between groups (involved patients – non-dominant controls; involved ACL-IB – involved ACL-R) were calculated.

Parameter	*ACL-IB*		*ACL-R*		*Controls*		*leg difference within a group*			*leg difference between groups (involved patients and non-dominant controls)*		
	Involved	*Uninvolved*	*Involved*	*Uninvolved*	*Non-dominant*	*Dominant*	*ACL-IB*	*ACL-R*	*Controls*	*ACL-IB vs Controls*	*ACL-R vs Controls*	*ACL-IB vs ACL-R*
	mean (SD)	*mean (SD)*	*mean (SD)*	*mean (SD)*	*mean (SD)*	*mean (SD)*	*95% CI*	*95% CI*	*95% CI*	*95% CI*	*95% CI*	*95% CI*
**SPATIO-TEMPORAL PARAMETER**												
Step time (s)	0.54 (0.01)	0.55 (0.02)	0.53 (0.01)	0.53 (0.01)	0.54 (0.01)	0.53 (0.01)	[-0.01;0.01]	[-0.01;0.00]	[-0.00;0.01]	[-0.01;0.03]	[-0.03;0.02]	[-0.01;0.0]
Step length (m)	0.75 (0.01)	0.74 (0.02)	0.74 (0.02)	0.74 (0.01)	0.75 (0.02)	0.76 (0.02)	[-0.01;0.02]	[-0.02;0.02]	[-0.01;0.01]	[-0.05;0.04]	[-0.06;0.03]	[-0.04;0.1]
Stance phase (%GC)	61.5 (0.7)	61.3 (4.0)	61.7 (0.5)	61.4 (0.3)	61.1 (0.3)	61.1 (0.3)	[-0.7;1.0]	[-0.1;0.6]	[-0.3;0.2]	[-0.4;1.2]	[-0.3;1.4]	[-1.0;0.7]
Single support phase (%GC)	39.1 (0.5)	39.3 (1.9)	39.0 (0.4)	38.9 (0.5)	39.0 (0.4)	39.1 (0.4)	[-0.8;0.4]	[-0.3;0.5]	[-0.5;0.5]	[-0.9;0.9]	[-1.0;0.8]	[-0.8;1.0]
Double support phase (%GC)	22.4 (0.6)	23.3 (7.4)	22.7 (0.4)	22.5 (0.6)	22.0 (0.4)	22.1 (0.4)	[-2.5;0.7]	[-0.2;0.5]	[-0.4;0.4]	[-1.1;1.9]	[-0.9;2.2]	[-1.8;1.3]
**GROUND REACTION FORCE**												
Medial (%BW)												
Minimum, GC	-3.9 (0.3)	-4.3 (0.4)	-3.8 (0.3)	-4.2 (0.3)	-4.6 (0.3)	-4.3 (0.4)	[-0.0;0.8]	[-0.1;0.9]	[-0.8;0.2]	[-0.5;2.0]	[-0.5;2.1]	[-1.3;1.2]
First maximum, ST	5.4 (0.6)	5.4 (0.5)	5.9 (0.3)	5.8 (0.5)	5.5 (0.4)	5.9 (0.5)	[-0.6;0.5]	[-0.6;0.7]	**[-0.9;-0.0]***	[-1.1;0.9]	[-0.6;1.4]	[-1.5;0.5]
Second maximum, ST	4.3 (0.4)	4.3 (0.4)	4.7 (0.3)	4.8 (0.3)	4.7 (0.3)	4.3 (0.4)	[-0.4;0.5]	[-0.5;0.3]	**[0.1;0.7]***	[-1.6;0.8]	[-1.2;1.2]	[-1.6;0.8]
Anterior (%BW)												
Maximum, GC	23.5 (0.7)	23.2 (0.9)	23.8 (0.6)	24.0 (0.6)	23.3 (0.8)	23.6 (0.8)	[-0.5;1.0]	[-0.7;0.5]	[-1.1;0.4]	[-2.2;2.5]	[-1.9;2.9]	[-2.7;2.1]
Minimum, GC	-20.2 (0.6)	-21.5 (0.8)	-21.5 (0.8)	-21.8 (0.6)	-21.2 (0.6)	-20.9 (0.7)	**[0.4;2.1]***	[-0.5;1.2]	[-1.0;0.3]	[-1.3;3.4]	[-2.5;2.3]	[-1.2;3.6]
Vertical (%BW)												
First maximum, ST	113.7 (1.5)	115.5 (1.8)	116.0 (1.3)	117.9 (1.4)	114.5 (1.2)	113.3 (1.2)	[-4.2;0.6]	[-3.7;-0.2]*	[-0.2;2.6]	[-5.6;4.0]	[-3.4;6.4]	[-7.2;2.6]
Second maximum, ST	111.5 (0.7)	112.9 (1.3)	113.5 (0.9)	115.1 (0.9)	114.9 (1.3)	114.6 (1.1)	[-3.0;0.2]	[-3.3;0.0]	[-1.0;1.6]	[-7.8;0.9]	[-5.8;3.1]	[-6.5;2.4]
**HIP**												
Flexion angle (°)												
Maximum, ST	40.9 (0.6)	40.5 (0.8)	38.9 (0.7)	38.4 (0.6)	37.7 (0.6)	39.7 (0.5)	[-2.1;2.8]	[-1.1;2.1]	**[-3.4;-0.6]***	[-2.0;8.4]	[-4.1;6.4]	[-3.3;7.3]
Minimum, GC	-3.0 (0.3)	-3.8 (0.5)	-5.8 (0.4)	-5.3 (0.3)	-4.7 (0.3)	-4.8 (0.5)	[-0.9;2.6]	[-1.9;0.9]	[-1.2;1.3]	[-3.2;6.7]	[-6.2;4.0]	[-2.2;7.9]
Maximum, SW	43.2 (0.8)	42.7 (0.7)	39.6 (0.6)	39.7 (0.5)	40.0 (0.4)	41.5 (0.5)	[-1.8;2.7]	[-1.6;1.3]	**[-2.9;-0.3]***	[-1.5;7.9]	[-5.2;4.4]	[-1.2;8.3]
Adduction angle (°)												
Mean, ST	-8.9 (0.3)	-9.2 (0.9)	-8.1 (0.3)	-8.8 (0.3)	-7.6 (0.2)	-7.2 (0.2)	[-1.2;1.8]	[-0.6;2.0]	[-1.7;0.9]	[-3.2;0.6]	[-2.5;1.4]	[-2.7;1.2]
Mean, SW	-1.8 (0.7)	-1.9 (0.3)	-1.2 (0.3)	-1.8 (0.2)	-1.2 (0.3)	-0.7 (0.2)	[-1.6;1.8]	[-0.6;1.9]	[-1.9;0.9]	[-2.5;1.4]	[-1.9;2.0]	[-2.6;1.4]
Internal rotation (°)												
Mean, GC	1.9 (0.4)	4.9 (0.5)	2.9 (0.3)	4.2 (0.3)	2.7 (0.1)	1.3 (0.2)	**[-5.3;-0.7]***	[-3.8;1.3]	[-1.4;4.3]	[-4.5;2.8]	[-3.5;3.9]	[-4.7;2.7]
Flexion moment (Nm/kg)												
Maximum, ST	0.74 (0.03)	0.74 (0.04)	0.77 (0.03)	0.77 (0.04)	0.82 (0.03)	0.80 (0.05)	[-0.04;0.06]	[-0.04;0.03]	[-0.02;0.05]	[-0.22;0.07]	[-0.19;0.10]	[-0.17;0.1]
Minimum, ST	-0.83 (0.02)	-0.86 (0.02)	-0.86 (0.02)	-0.93 (0.02)	-0.97 (0.03)	-0.97 (0.03)	[-0.01;0.07]	**[0.03;0.10]***	[-0.05;0.05]	**[0.01;0.27]***	[-0.03;0.23]	[-0.09;0.2]
Adduction moment (Nm/kg)												
First minimum, ST	-0.07 (0.02)	-0.07 (0.02)	-0.09 (0.02)	-0.10 (0.02)	-0.17 (0.03)	-0.16 (0.03)	[-0.02;0.04]	[-0.02;0.04]	[-0.04;0.03]	**[0.04;0.17]***	**[0.01;0.14]***	[-0.04;0.1]
First maximum, ST	0.89 (0.03)	0.87 (0.04)	0.85 (0.02)	0.85 (0.03)	0.80 (0.03)	0.80 (0.04)	[-0.03;0.06]	[-0.05;0.04]	[-0.06;0.05]	[-0.01;0.19]	[-0.05;0.15]	[-0.06;0.1]
Second maximum, ST	0.77 (0.03)	0.76 (0.04)	0.74 (0.03)	0.78 (0.03)	0.73 (0.04)	0.68 (0.03)	[-0.03;0.06]	[-0.09;0.01]	[-0.02;0.10]	[-0.05;0.14]	[-0.09;0.11]	[-0.07;0.1]
Internal rotation moment (Nm/kg)												
Maximum, GC	0.12 (0.01)	0.15 (0.02)	0.11 (0.01)	0.14 (0.01)	0.15 (0.01)	0.14 (0.01)	**[-0.04;-0.01]***	**[-0.04;-0.02]***	**[0.01;0.03]***	**[-0.06;-0.00]***	**[-0.07;-0.01]***	[-0.02;0.0]
Power (W/kg)												
Maximum, GC	1.28 (0.06)	1.37 (0.05)	1.29 (0.05)	1.34 (0.05)	1.34 (0.05)	1.33 (0.08)	**[-0.17;-0.02]***	[-0.15;0.05]	[-0.05;0.07]	[-0.28;0.16]	[-0.27;0.18]	[-0.24;0.2]
**KNEE**												
Flexion angle (°)												
Maximum, ST	22.4 (0.8)	22.3 (0.9)	21.9 (0.6)	23.0 (0.8)	22.0 (0.7)	23.0 (0.7)	[-2.1;2.2]	[-2.7;0.4]	[-2.3;0.4]	[-2.9;3.8]	[-3.5;3.3]	[-2.9;3.9]
Minimum, ST	9.3 (0.6)	6.9 (0.7)	7.5 (0.6)	6.2 (0.7)	6.4 (0.5)	7.9 (0.4)	**[0.5;4.4]***	[-0.3;2.9]	**[-2.9;-0.1]***	[-0.7;6.5]	[-2.5;4.7]	[-1.8;5.4]
Maximum, SW	72.4 (0.5)	71.2 (0.4)	68.6 (0.9)	69.4 (0.6)	69.7 (0.6)	71.2 (0.5)	[-1.2;3.5]	[-2.6;1.1]	**[-2.8;-0.3]***	[-0.4;5.9]	[-4.2;2.2]	**[0.5;7.0]***

Parameter	ACL-IB		ACL-R		Controls		leg difference within a group			leg difference between groups (involved patients and non-dominant controls)		
	Involved *mean (SD)*	*Uninvolved* *mean (SD)*	*Involved* *mean (SD)*	*Uninvolved* *mean (SD)*	*Non-dominant* *mean (SD)*	*Dominant* *mean (SD)*	*ACL-IB* *95% CI*	*ACL-R* *95% CI*	*Controls* *95% CI*	*ACL-IB vs Controls* *95% CI*	*ACL-R vs Controls* *95% CI*	*ACL-IB vs ACL-R* *95% CI*
Abduction angle (°)												
Mean, ST	3.2 (0.2)	4.2 (0.2)	4.0 (0.2)	4.7 (0.2)	2.6 (0.1)	1.9 (0.1)	[-2.1;0.0]	[-1.8;0.4]	[-0.6;2.0]	[-1.5;2.6]	[-0.8;3.5]	[-2.9;1.3]
Internal rotation angle (°)												
Mean, ST	-0.1 (0.5)	-0.8 (0.3)	-0.0 (0.4)	-2.5 (0.4)	-0.8 (0.4)	-0.9 (0.4)	[-0.9;2.3]	**[0.4;4.5]***	[-1.2;1.3]	[-1.7;3.1]	[-1.6;3.2]	[-2.5;2.4]
Mean, SW	7.7 (0.5)	6.7 (0.8)	7.1 (0.5)	4.5 (0.7)	5.7 (0.3)	5.8 (0.3)	[-0.4;2.4]	**[0.7;4.4]***	[-1.3;1.2]	[-0.9;4.9]	[-1.6;4.3]	[-2.3;3.6]
Anterior tibia position (mm)												
Maximum, GC	16.9 (0.9)	15.1 (1.2)	17.3 (1.0)	20.4 (0.9)	23.0 (0.7)	22.5 (1.0)	[-1.8;5.4]	[-6.9;0.8]	[-2.6;3.6]	**[-11.6;-0.7]***	**[-11.3;-0.2]***	[-6.0;5.1]
Flexion moment (Nm/kg)												
Maximum, ST	0.66 (0.04)	0.73 (0.05)	0.76 (0.03)	0.81 (0.03)	0.75 (0.03)	0.76 (0.05)	**[-0.13;-0.00]***	**[-0.11;-0.00]***	[-0.06;0.05]	[-0.25;0.08]	[-0.17;0.17]	[-0.26;0.1]
Minimum, second half ST	-0.26 (0.02)	-0.31 (0.03)	-0.24 (0.02)	-0.30 (0.02)	-0.36 (0.02)	-0.35 (0.02)	[-0.00;0.11]	**[0.01;0.11]***	[-0.06;0.03]	[-0.00;0.20]	**[0.02;0.23]***	[-0.13;0.1]
Adduction moment (Nm/kg)												
First maximum, ST	0.51 (0.03)	0.48 (0.03)	0.47 (0.02)	0.45 (0.02)	0.49 (0.02)	0.54 (0.03)	[-0.01;0.06]	[-0.02;0.07]	**[-0.08;-0.01]***	[-0.07;0.10]	[-0.10;0.07]	[-0.05;0.1]
Second maximum ST	0.39 (0.02)	0.39 (0.02)	0.36 (0.02)	0.36 (0.02)	0.40 (0.02)	0.41 (0.02)	[-0.04;0.03]	[-0.03;0.04]	[-0.05;0.02]	[-0.08;0.07]	[-0.11;0.05]	[-0.05;0.1]
Internal rotation moment (Nm/kg)												
Maximum, ST	0.17 (0.01)	0.18 (0.01)	0.16 (0.01)	0.17 (0.01)	0.19 (0.01)	0.18 (0.01)	[-0.03;0.01]	**[-0.02;-0.00]***	[-0.00;0.02]	[-0.05;0.01]	**[-0.06;-0.00]***	[-0.02;0.0]
Power (W/kg)												
Maximum, ST	1.11 (0.09)	1.25 (0.12)	1.17 (0.05)	1.46 (0.07)	1.36 (0.08)	1.34 (0.14)	[-0.32;0.03]	**[-0.43;-0.14]***	[-0.12;0.16]	[-0.55;0.05]	[-0.49;0.12]	[-0.37;0.2]
Minimum, ST	-1.28 (0.14)	-1.43 (0.19)	-1.38 (0.09)	-1.66 (0.15)	-1.51 (0.12)	-1.51 (0.22)	[-0.00;0.29]	**[0.14;0.41]***	[-0.16;0.16]	[-0.18;0.64]	[-0.29;0.55]	[-0.32;0.5]
Minimum, SW	-2.22 (0.09)	-2.26 (0.08)	-2.24 (0.06)	-2.38 (0.08)	-2.45 (0.14)	-2.38 (0.11)	[-0.09;0.14]	**[0.02;0.25]***	[-0.19;0.05]	[-0.16;0.62]	[-0.19;0.59]	[-0.37;0.4]
**ANKLE**												
Dorsiflexion angle (°)												
Maximum, GC	14.7 (0.5)	14.1 (1.2)	14.9 (0.5)	14.7 (0.7)	14.2 (0.3)	14.8 (0.3)	[-0.3;1.8]	[-0.9;1.2]	[-1.6;0.4]	[-1.6;2.5]	[-1.5;2.7]	[-2.3;1.9]
Minimum, GC	-22.3 (0.8)	-22.7 (0.9)	-20.8 (0.9)	-20.1 (1.2)	-20.9 (0.9)	-21.2 (0.9)	[-0.9;2.2]	[-2.5;1.1]	[-1.4;2.1]	[-5.0;2.2]	[-3.5;3.7]	[-5.2;2.1]
Dorsiflexion moment (Nm/kg)												
Maximum, ST	1.52 (0.03)	1.53 (0.03)	1.53 (0.02)	1.55 (0.04)	1.57 (0.03)	1.56 (0.02)	[-0.05;0.03]	[-0.08;0.04]	[-0.03;0.04]	[-0.17;0.06]	[-0.16;0.07]	[-0.13;0.1]
Power (W/kg)												
Maximum, GC	3.85 (0.14)	3.88 (0.22)	3.88 (0.12)	3.94 (0.14)	4.15 (0.16)	4.17 (0.16)	[-0.23;0.17]	[-0.26;0.14]	[-0.25;0.21]	[-0.94;0.34]	[-0.92;0.39]	[-0.68;0.6]
**MUSCLE ACTIVITY**												
Peak activation during gait												
Semitendinosus (%MVC)	22.0 (2.6)	22.9 (2.7)	30.7 (4.3)	15.6 (1.6)	20.0 (2.7)	21.1 (2.4)	[-7.6;5.9]	**[6.4;23.5]***	[-3.1;2.8]	[-8.3;12.3]	**[0.1;21.3]***	[-19.2;1.8]
Vastus Medialis (%MVC)	17.4 (3.2)	17.7 (2.7)	22.4 (8.5)	20.6 (6.2)	18.4 (2.6)	22.2 (5.3)	[-3.8;3.3]	[-8.5;12.6]	[-9.0;1.3]	[-10.6;8.7]	[-5.9;13.8]	[-14.8;4.9]
Vastus Lateralis (%MVC)	32.1 (13.0)	25.9 (9.7)	23.0 (5.8)	21.6 (5.4)	25.6 (5.7)	26.6 (14.4)	[-2.7;6.9]	[-7.4;3.8]	[-6.2;0.8]	[-13.7;26.7]	[-22.8;17.7]	[-11.5;29.6]
Gastrocnemius Medialis (%MCW)	81.7 (3.8)	80.4 (7.4)	80.4 (3.9)	81.8 (5.5)	81.3 (3.1)	84.0 (4.0)	[-2.7;6.9]	[-7.4;3.8]	[-6.2;0.8]	[-3.7;4.6]	[-5.3;3.5]	[-3.1;5.8]
Gluteus Medius (%MCW)	82.5 (3.2)	82.1 (2.8)	83.8 (4.1)	84.4 (5.2)	82.4 (4.0)	81.1 (3.2)	[-3.7;3.9]	[-3.3;4.0]	[-5.8;2.4]	[-4.2;4.6]	[-3.0;5.9]	[-5.8;3.2]
Tibialis Anterior (%MCW)	83.2 (2.8)	85.8 (3.8)	83.4 (4.0)	83.1 (4.5)	84.2 (2.9)	84.1 (3.5)	[-5.9;0.9]	[-2.8;3.4]	[-2.3;2.5]	[-4.6;2.6]	[-4.5;2.8]	[-3.8;3.5]

Abbreviation: SD, standard deviation; CI, confidence Interval; BW, body weight; GC: gait cycle; ST, stance phase; SW, swing phase; MVC, isokinetic maximum voluntary contraction; MCW, maximum contraction during walking. *95%CI is excluding zero, indicating a significant difference. Values with grey shaded area have been reported in the linked manuscript and are presented here twice for completeness and comparability.

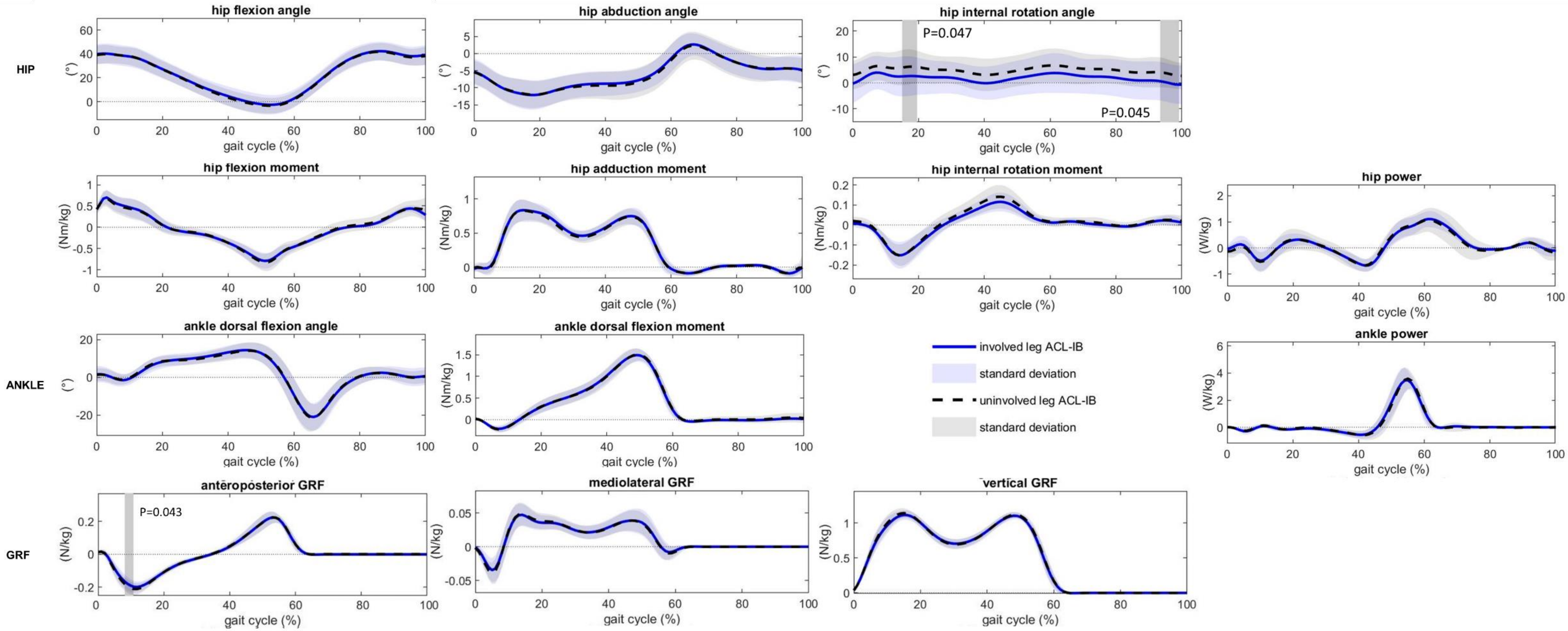

Supplement Figure S1: Mean (1 standard deviation) trajectories of hip and ankle biomechanics (kinematics and kinetics) and ground reaction force (GRF as % body weight (BW)) of the involved and uninvolved leg in patients 2 years after anterior cruciate ligament repair and InternalBrace^{TM} augmentation (ACL-IB), including significant different intervals in side-to-side differences (grey area) using statistical parametric mapping analysis (paired t-test, P<0.05).

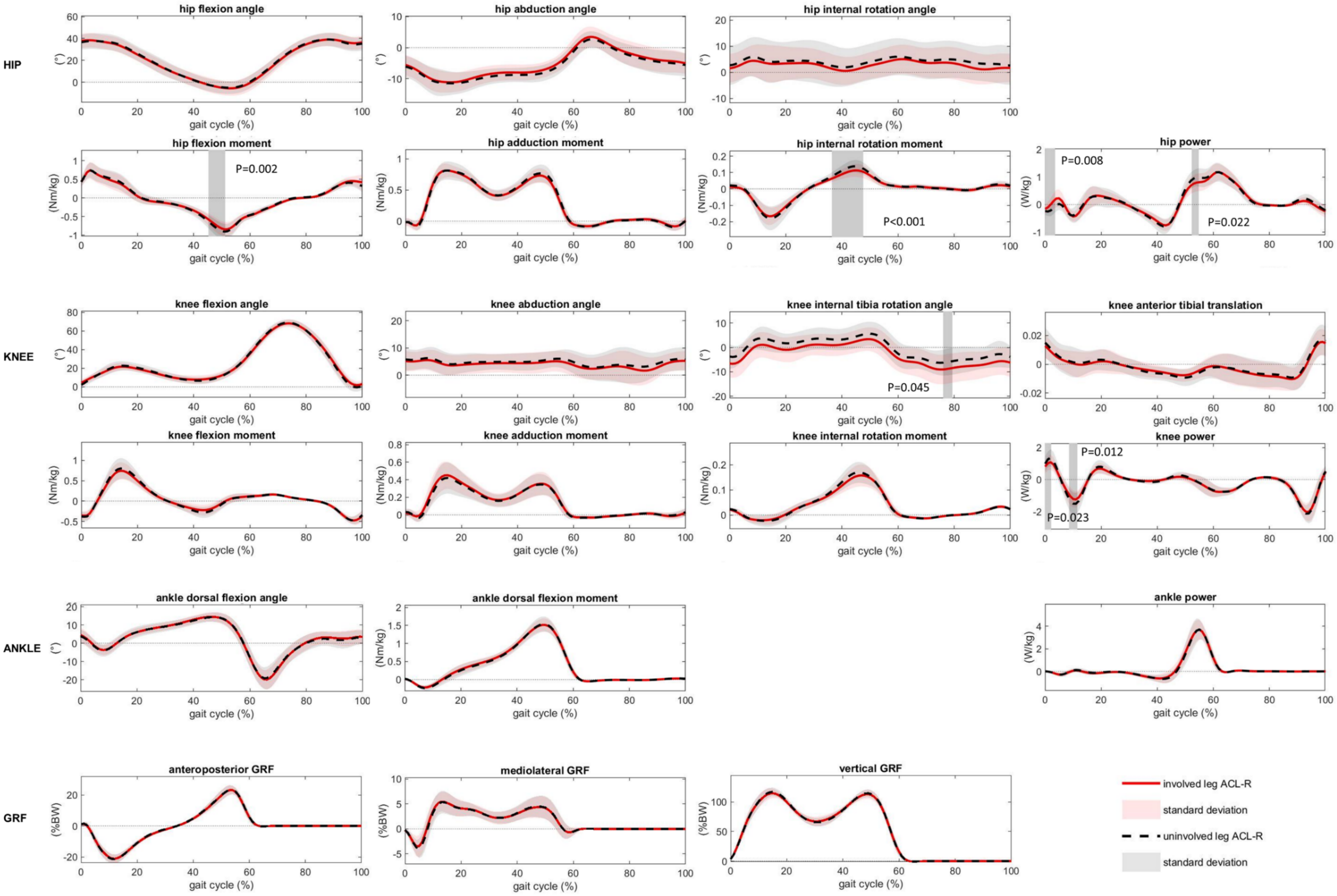
HIP
KNEE
ANKLE
GRF
hip flexion angle
hip abduction angle
hip internal rotation angle
hip flexion moment
P=0.002
hip adduction moment
hip internal rotation moment
P<0.001
hip power
P=0.008
P=0.022
knee flexion angle
knee abduction angle
knee internal tibia rotation angle
P=0.045
knee anterior tibial translation
knee flexion moment
knee adduction moment
knee internal rotation moment
knee power
P=0.012
P=0.023
ankle dorsal flexion angle
ankle dorsal flexion moment
ankle power
anteroposterior GRF
mediolateral GRF
vertical GRF
gait cycle (%)
(°)
(Nm/kg)
(W/kg)
(%BW)
involved leg ACL-R
standard deviation
uninvolved leg ACL-R
standard deviation

Supplement Figure S2: Mean (1 standard deviation) trajectories of hip, knee and ankle biomechanics (kinematics and kinetics) and ground reaction force (GRF as % body weight (BW)) of the involved and uninvolved leg in patients 2 years after anterior cruciate ligament reconstruction (ACL-R), including significant different intervals in side-to-side differences (grey area) using statistical parametric mapping analysis (paired t-test, $P<0.05$).

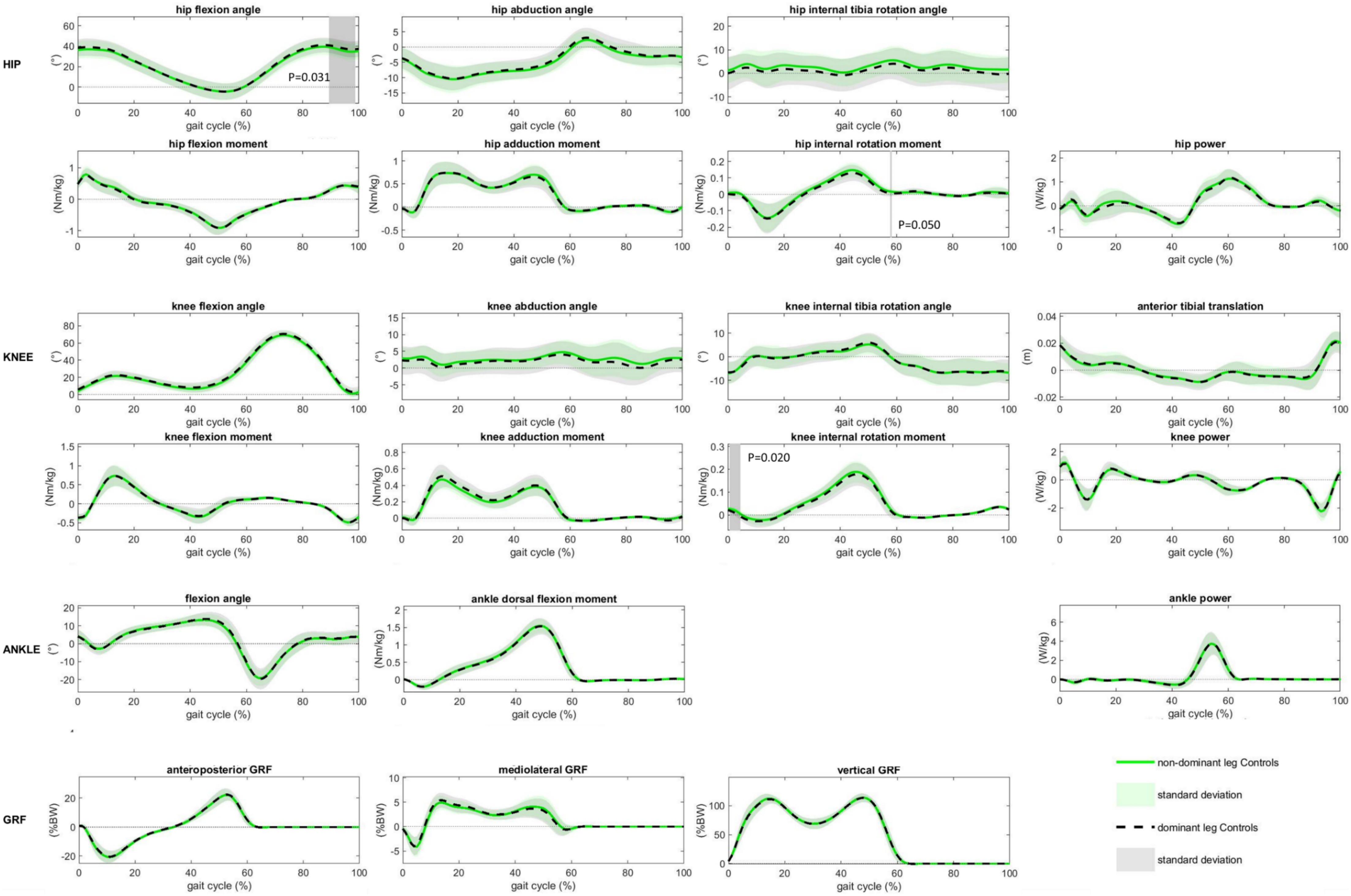
HIP
hip flexion angle
(°)
P=0.031
gait cycle (%)
hip abduction angle
(°)
gait cycle (%)
hip internal tibia rotation angle
(°)
gait cycle (%)
hip flexion moment
(Nm/kg)
gait cycle (%)
hip adduction moment
(Nm/kg)
gait cycle (%)
hip internal rotation moment
(Nm/kg)
P=0.050
gait cycle (%)
hip power
(W/kg)
gait cycle (%)
KNEE
knee flexion angle
(°)
gait cycle (%)
knee abduction angle
(°)
gait cycle (%)
knee internal tibia rotation angle
(°)
gait cycle (%)
anterior tibial translation
(m)
gait cycle (%)
knee flexion moment
(Nm/kg)
gait cycle (%)
knee adduction moment
(Nm/kg)
gait cycle (%)
knee internal rotation moment
(Nm/kg)
P=0.020
gait cycle (%)
knee power
(W/kg)
gait cycle (%)
ANKLE
flexion angle
(°)
gait cycle (%)
ankle dorsal flexion moment
(Nm/kg)
gait cycle (%)
ankle power
(W/kg)
gait cycle (%)
GRF
anteroposterior GRF
(%BW)
gait cycle (%)
mediolateral GRF
(%BW)
gait cycle (%)
vertical GRF
(%BW)
gait cycle (%)
non-dominant leg Controls
standard deviation
dominant leg Controls
standard deviation

Supplement Figure S3: Mean (1 standard deviation) trajectories of hip, knee and ankle biomechanics and ground reaction force (GRF as % of body weight (BW)) of the non-dominant and dominant leg (personal subjective rating) in healthy controls, including significant different intervals in side-to-side differences (grey area) using statistical parametric mapping analysis (paired t-test, $P<0.05$).

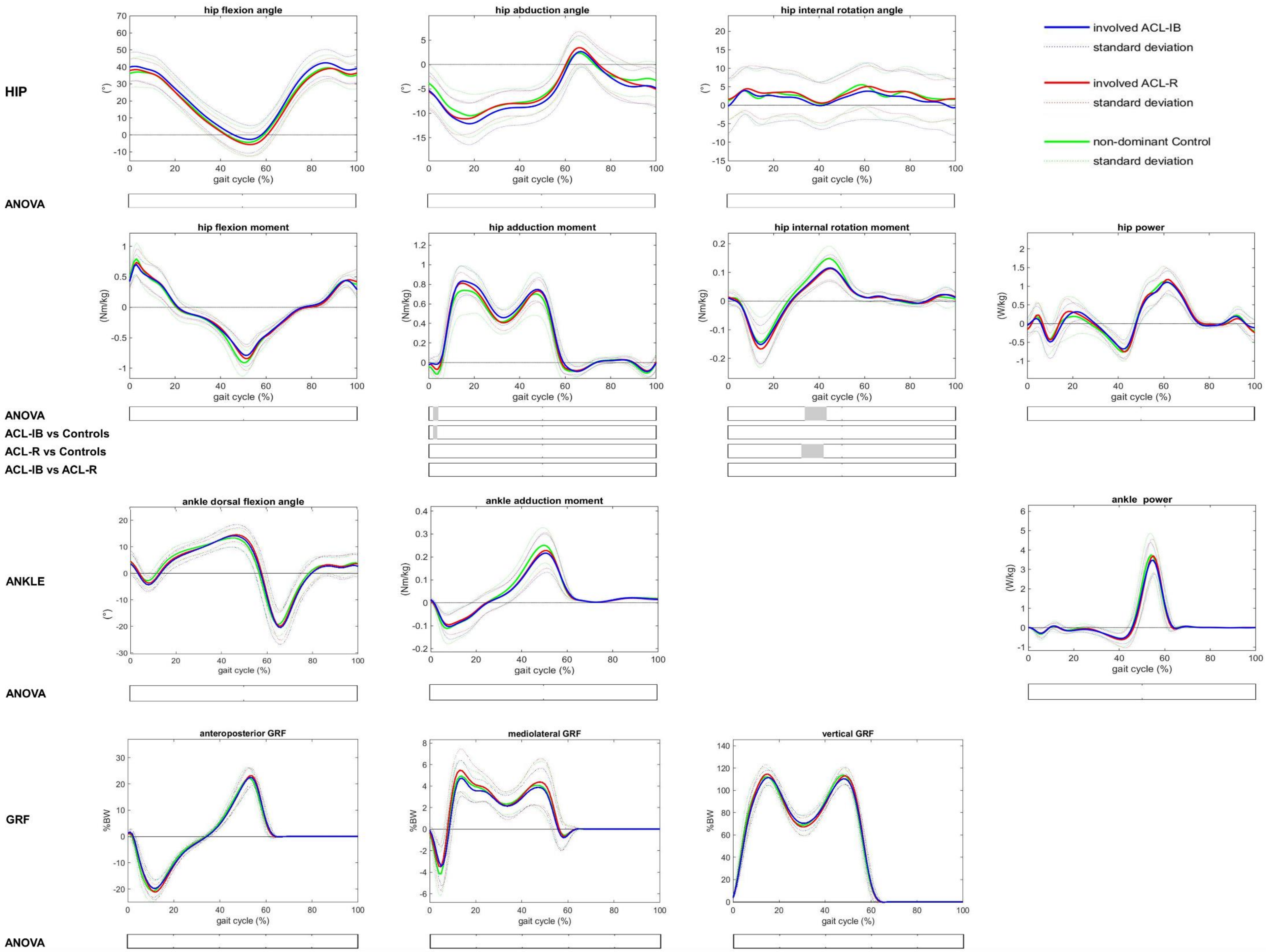
HIP
hip flexion angle
hip abduction angle
hip internal rotation angle
involved ACL-IB
standard deviation
involved ACL-R
standard deviation
non-dominant Control
standard deviation
(°)
gait cycle (%)
ANOVA
hip flexion moment
hip adduction moment
hip internal rotation moment
hip power
(Nm/kg)
(W/kg)
ANOVA
ACL-IB vs Controls
ACL-R vs Controls
ACL-IB vs ACL-R
ANKLE
ankle dorsal flexion angle
ankle adduction moment
ankle power
ANOVA
GRF
anteroposterior GRF
mediolateral GRF
vertical GRF
%BW
ANOVA

Supplement Figure S4: Comparison of mean (1 standard deviation) trajectories of hip and ankle biomechanics (kinematics and kinetic), and ground reaction force (GRF as % body weight (BW)) in the involved leg of patients 2 years after anterior cruciate ligament repair and InternalBrace^{TM} augmentation (ACL-IB), patients 2 years after ACL reconstruction (ACL-R) and the non-dominant leg of healthy controls using statistical parametric mapping analysis (one-way analysis of variance and Bonferroni post hoc test, P<0.05). Gray area indicates significant different intervals.

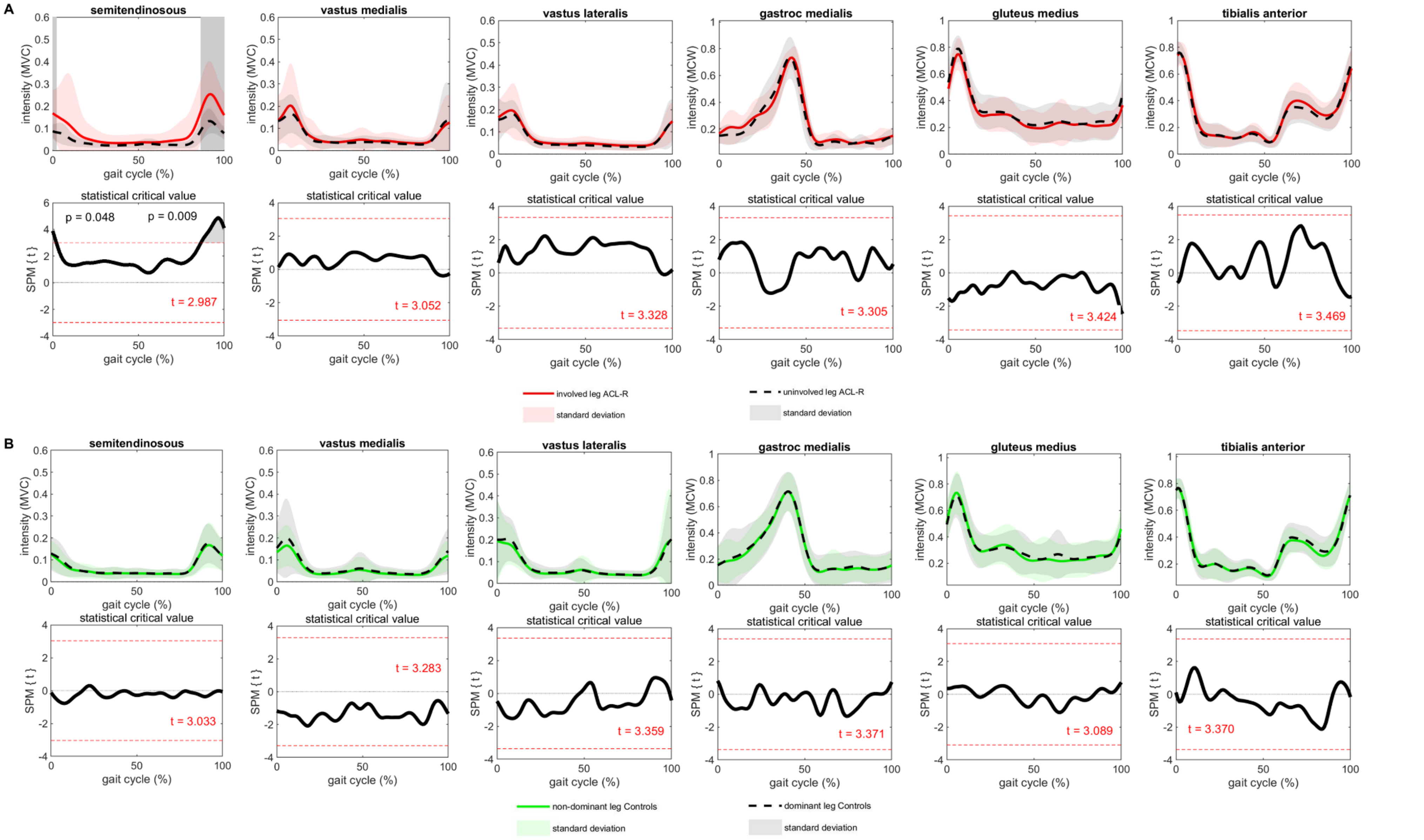

Supplement Figure S5: Mean (1 standard deviation) trajectories of activity of lower leg muscles and statistical parametric mapping analysis (t-test, P<0.05) between the legs of patients 2 years after anterior cruciate ligament reconstruction (ACL-R, A) and healthy controls (B). Amplitudes were normalized to maximum voluntary isokinetic contraction (MVC) or to maximal activity during walking cycle for each leg (maximum contraction during walking, MCW). Gray area indicates significant different intervals.
